# An *In Silico* Analysis of the Binding Modes and Binding Affinities of Small Molecule Modulators of PDZ-Peptide Interactions

**DOI:** 10.1371/journal.pone.0071340

**Published:** 2013-08-08

**Authors:** Garima Tiwari, Debasisa Mohanty

**Affiliations:** Bioinformatics Center, National Institute of Immunology, Aruna Asaf Ali Marg, New Delhi, India; University of Edinburgh, United Kingdom

## Abstract

Inhibitors of PDZ-peptide interactions have important implications in a variety of biological processes including treatment of cancer and Parkinson’s disease. Even though experimental studies have reported characterization of peptidomimetic inhibitors of PDZ-peptide interactions, the binding modes for most of them have not been characterized by structural studies. In this study we have attempted to understand the structural basis of the small molecule-PDZ interactions by *in silico* analysis of the binding modes and binding affinities of a set of 38 small molecules with known K_i_ or K_d_ values for PDZ2 and PDZ3 domains of PSD-95 protein. These two PDZ domains show differential selectivity for these compounds despite having a high degree of sequence similarity and almost identical peptide binding pockets. Optimum binding modes for these ligands for PDZ2 and PDZ3 domains were identified by using a novel combination of semi-flexible docking and explicit solvent molecular dynamics (MD) simulations. Analysis of the binding modes revealed most of the peptidomimectic ligands which had high K_i_ or K_d_ moved away from the peptide binding pocket, while ligands with high binding affinities remained in the peptide binding pocket. The differential specificities of the PDZ2 and PDZ3 domains primarily arise from differences in the conformation of the loop connecting βB and βC strands, because this loop interacts with the N-terminal chemical moieties of the ligands. We have also computed the MM/PBSA binding free energy values for these 38 compounds with both the PDZ domains from multiple 5 ns MD trajectories on each complex *i.e.* a total of 228 MD trajectories of 5 ns length each. Interestingly, computational binding free energies show good agreement with experimental binding free energies with a correlation coefficient of approximately 0.6. Thus our study demonstrates that combined use of docking and MD simulations can help in identification of potent inhibitors of PDZ-peptide complexes.

## Introduction

Understanding the molecular basis of the interactions involving various PRMs (Peptide Recognition Modules) is crucial not only for deciphering protein interaction networks, but also for potential therapeutic applications involving modulation of key interaction interfaces using small molecules. The transient nature of protein-protein interactions mediated by PRMs and involvement of a small part of a polypeptide chain makes it amenable for disruption using small molecules. In this respect, small domains present in various proteins with different biological functions and recurrent in protein-protein interactions are the ones, which draw special attention. PSD-95/discs-large/ZO-1 (PDZ) protein domains are one of the highly abundant domains in human proteome [1]. PDZ domains are relatively small domains of approximately 100 amino acids and bind with the extreme C-terminal of their interacting partners [2], [3]. The structure of PDZ domain consists of five to six β-strands (βA- βF) and two α-helices (αA and αB). The C-terminus of the interaction partner binds as an anti parallel β-strand in a groove between βB strand and the αB helix. PDZ domain containing proteins are mostly involved in trafficking, recruiting and assembling of intracellular enzymes and membrane receptors into signaling transduction complexes. PDZ domains are known to increase the specificity and efficiency of intracellular interaction network of important PPIs downstream of receptor activation involving various signaling enzymes [4], [5].

The association of PDZ-domain containing proteins in various diseases like cancer [6]–[8], cystic fibrosis [9], [10], schizophrenia [11], Parkinson’s disease [12], Alzheimer’s disease [13], cerebral ischemia [14], pain [15], [16] and disorders in the central nervous system makes it a putative target for development of drugs [17]–[19]. Various peptide and non-peptide small molecules have been developed as inhibitors of PPIs mediated by PDZ domains [16], [20]. PDZ domains have been considered as difficult targets for small molecule inhibitors, because of the shallow and elongated binding pocket. However, there are some reports available that suggest that small molecule inhibitors indeed bind to PDZ domains like Disheveled with affinities approximately 10 µM [21]–[23]. Due to this reason, the peptide backbone has been exploited extensively to develop potent inhibitors of PDZ domains. Peptide based strategies including cyclization of hexapeptide and dimerization of peptide ligands have been used till date for inhibitor development [24]–[26]. A recent example is a 20-mer linear peptide Tat-N2B (NA-1, YGRKKRRQRRR-KLSSIESDV) [14], which has gone under phase-II clinical trial as a putative neuroprotective drug related to stroke and endovascular procedures. Attempts have also been made to develop inhibitors with reduced size and increased potency for disrupting PSD-95/NMDA receptor interaction. Bach *et al* have used C-terminal region of GluN2B protein (YEKLSSIESDV) as a template for developing peptidomimetic inhibitors and have demonstrated that N-alkylation of tetrapeptides improved the affinity up to 40 fold. They have identified N-cyclohexylethylETAV as a potent inhibitor of PDZ2 of PSD-95 protein [27].

Even though several experimental studies [28] have reported development and characterization of small molecule or peptidomimetic inhibitors of PDZ-peptide interactions, the binding modes for most of them have not been characterized by structural studies. In absence of any structural or computational analysis of the binding modes, it is difficult to understand the structural basis of the differential binding affinities of the various small molecules to the same PDZ domain. Similarly, it is also necessary to understand, how PDZ2 and PDZ3 of PSD-95 show differential selectivity for the same small molecule despite having a high degree of sequence similarity and almost identical peptide binding pocket residues. Understanding of the structural basis of the small molecule-PDZ interactions is crucial for *in silico* design of novel and highly potent PDZ inhibitors. Therefore, in this study an attempt has been made to predict the binding modes for a set of experimentally characterized small molecule/peptidomimetic inhibitors of PDZ domains and investigate whether the computed binding free energy values correlate with the experimentally determined binding affinities. Binding modes have been identified by using a combination of docking and explicit solvent molecular dynamics (MD) simulations and binding free energies have been computed by MM/PBSA approach. The inhibitor dataset chosen for this analysis consists of a set of 38 peptidomimetic inhibitors for which experimental binding affinity values are known for PDZ2 and PDZ3 domains of PSD-95 [29].

## Materials and Methods

### Compilation of Known Small Molecule Inhibitors of PDZ Domains

An extensive survey of published literature on structure, function and interaction partners of PDZ domains was carried out to find out the chemical structures of known inhibitors of PDZ domains. This resulted in a total of 60 small molecules or peptidomimetic compounds that had been shown in experimental studies to inhibit the interaction of various PDZ domains with their native peptide ligands. The chemical structures of most of these 60 small molecules were retrieved from PubChem [30] as flat files in SDF format and in cases where SDF files were not available, the molecules were drawn using ChemDraw software [31] and chemical connectivity of the compounds were saved as MOL files. The SDF and MOL files were given as input to the HCLUST module of R package [32] for clustering these 60 molecules based on the similarities in their chemical structures.

### Docking Studies for Modeling PDZ-inhibitor Complexes

**Figure 1:**
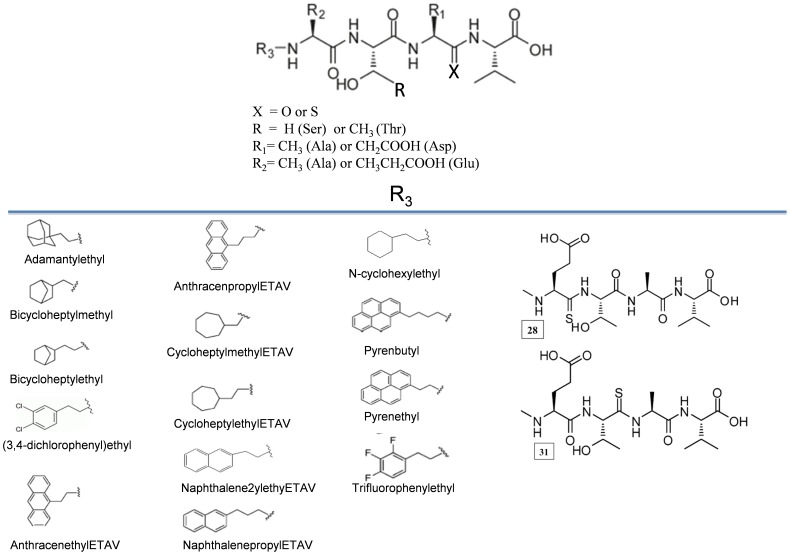
Tetrapeptide backbone modifications. Figure showing various modifications done to the N-terminal of the tetrapeptides for obtaining the peptidomimetics. In molecules 28 and 31, amide bonds are replaced with thioamide bonds between Glu-Thr and Thr-Ala of N-methylated ETAV, respectively.

**Figure S1** shows the protocol followed for exploring the various binding modes in which a ligand can bind to the PDZ domain. The steps shown in the workflow have been discussed in later sections. Out of total 60 known inhibitors of PDZ domain, a dataset of 38 ligands, which were experimentally characterized in terms of binding specificity towards two PDZ domains *i.e.* PDZ2 and PDZ3 of PSD-95 protein, was selected for docking analysis. **Figure 1** depicts the chemical structures of the 38 peptidomimetic inhibitors which have been obtained by various modifications done on the backbone of tetrapeptides. The SDF and MOL files representing the chemical connectivities of the inhibitors were given as input to babel program of OpenBabel 2.2.3 [33], [34] for generating the coordinates of the three dimensional structures of these small molecule inhibitors in PDB format. The coordinates of the small molecules were given as input to AUTODOCK VINA [35] for docking them onto crystal structures of PDZ2 *i.e.* 1QLC [36] and PDZ3 *i.e.* 1BE9 [37] domains of PSD-95. The peptide from 1BE9 was removed from the structure before docking. During docking the backbone φ, ψ dihedrals and χ^1^ sidechain dihedral were kept rotatable. The residues of PDZ domain, which were known to interact with the peptide from earlier studies, were made flexible during docking. The docking grid box of dimension 38Å × 74Å × 34Å was made which covered the entire PDZ domains. Docking was carried out using Broyden-Fletcher-Goldfarb-Shanno algorithm for local optimization [38]. The number of steps in a docking run is determined heuristically, depending on the size and flexibility of the ligand and the flexible side chains. However, the number of runs is set by the exhaustiveness parameter, which has been set to a maximum of 8 on the scale of 1–8 for thoroughness of search. Default values for other parameters like generations and energy evaluations were used.

### Molecular Dynamics Simulations on PDZ-inhibitor Complexes

Explicit solvent Molecular Dynamics (MD) simulations were performed on selected PDZ-inhibitor complexes obtained from docking studies using AMBER 9 [39]. The ff03 force field [40] was used to assign force field parameters for various atoms of different PDZ domains, which contained standard amino acid residues only. Since the small molecule inhibitors or peptidomimetics, which contained non-standard chemical moieties, the force field parameters for those chemical moieties were assigned using the Antechamber module of AMBER 9 [41]. While assigning force field parameters for the inhibitors using Antechamber, the partial charges were calculated by AM1-BCC method and bond length, bond angle, dihedral and van der Waals parameters were obtained by assigning GAFF atomtypes [42]. The PDZ-inhibitor complexes obtained from docking were first minimized in vacuum to 0.001 kcal/mol/Å as RMS gradient of potential energy using SANDER module of AMBER 9 to remove steric clashes, if any. The complexes were then solvated in a rectangular box containing TIP3P water model [43], with box boundaries 8Å away from the outermost protein atoms along x, y and z directions. The solvated complexes were also minimized by steepest descent algorithm using the convergence criteria of 0.001 kcal/mole/Å as RMS gradient of potential energy. After minimization, MD simulation was carried out using a time step of 1 fs and the temperature of the system was gradually increased to 300 K over a 50 ps simulation under NVT conditions and then simulations were carried out under NPT conditions at 1 atm pressure for another 50 ps. After the temperature and pressure equilibration process, the simulations for production dynamics were run for 5 ns under NVT conditions. Simulations were run using periodic boundary conditions. Particle Mesh Ewald [44] method was employed to calculate the long-range electrostatic interactions and non-bonded interactions were calculated using a distance cutoff of 8Å. For each PDZ-inhibitor complex, 3 independent 5 ns MD trajectories were computed starting from different random number seeds to assign different sets of initial velocities in each of these three simulations. The resulting MD trajectories were analyzed using PTRAJ module of AMBER package and Perl scripts developed in house.

### Analysis of Contacts between Inhibitors and PDZ Domains

Last 1ns of the 5 ns MD trajectories for different PDZ-inhibitor complexes were taken and snapshots at an interval of 50 ps were extracted resulting in 20 conformations of PDZ-inhibitor complexes for each 5 ns trajectory. The contacting residue pairs between the PDZ domain and the inhibitor were identified by using a distance cutoff of 3.5Å between any two atoms of the residue pair. Only those contacting residue pairs which were present in more than 50% of the PDZ-inhibitor complexes in last 1 ns of the MD trajectory were considered as residue pairs having stable interactions.

### Binding Free Energy Calculation using MM/PBSA Approach

Last 1 ns from each of the three 5 ns explicit solvent MD trajectories for different PDZ-inhibitor complexes were utilized for calculation of the binding free energies. A total of 100 conformations of PDZ-inhibitor complexes from each trajectory were obtained by collecting snapshots equally spaced at an interval of 10 ps. The binding free energy was calculated using implicit solvent MM/PBSA approach [45], by removing coordinates of solvent molecules from each of the extracted snapshots and only the coordinates of the PDZ domain and bound inhibitor were used for binding free energy calculation. The free energy was calculated for each molecular species namely, PDZ-inhibitor complex, receptor (PDZ domain) and ligand (inhibitor). The binding free energy for the inhibitor was computed by using the equation, ΔG_binding_ = G_complex_ − G_PDZ-domain_ – G_inhibitor_.

## Results

### Analysis of Structural Diversity within Known Inhibitors of PDZ Domains

**Figure 2:**
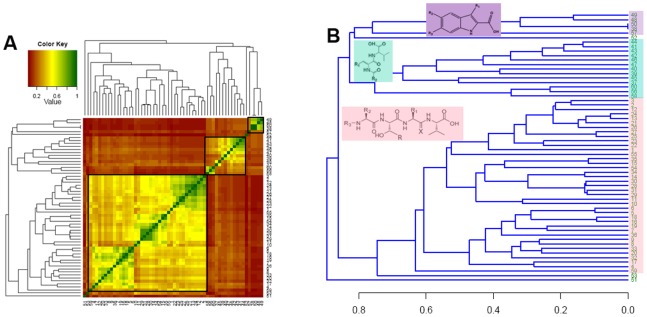
Clustering of PDZ modulator. (A) Heatmap depicting the clustering of known inhibitors of PDZ domains on the basis of their similarities in chemical structures. (B) Dendrogram depicting the clustering of known inhibitors of PDZ domains. Chemical structures of representative inhibitors from each cluster are also shown.

The experimentally characterized small molecule or peptidomimetic inhibitors of PDZ-peptide interactions were compiled based on literature survey. **Figure S2** shows in tabular format the chemical structures of the inhibitors, the name of the PDZ domain, which they inhibit, and whether the binding affinity in terms of K_i_ or K_d_ values is known. It may be noted that K_i_ and K_d_ both represent binding affinity and were measured in the same way, only different notation has been used in literature by the experimental groups which reported these inhibitors. As can be seen from **Figure S2**, only in case of six out of these 60 inhibitors binding affinity values are not known because they have been experimentally characterized by absence of protein-protein complex involving PDZ domain and the native substrate protein. For the remaining 54 inhibitors experimental K_i_ or K_d_ values have been reported based on thermodynamic experiments. In order to analyze the structural diversities of these 60 inhibitor molecules, they were clustered based on similarities in their chemical structures. **Figure 2A** shows the clustering of these 60 molecules in the form of a heatmap, while in **Figure 2B** the clustering is represented as a dendrogram. The heatmap as well as the dendrogram clearly depict that all the molecules under study can be categorized into 3 major groups. They essentially represent modifications on three distinct chemical scaffolds and **Figure 2B** shows representative structures from each of these three groups. However, the molecules 51, 52 and 53 did not fall in any of these three clusters. These 3 molecules have been characterized against different PDZ domains by NMR and biochemical studies [23], [28], [46], [47]. Out of the five molecules in the first cluster (purple colored), K_i_ value is known only for the compound number 57, which inhibits PDZ domain of Shank3 [48]. Molecule numbered 38, 48, 49 and 50 are known as inhibitors for 1^st^ PDZ domain of PSD-95 protein, but K_i_ or K_d_ values are not known for them. The binding of the inhibitors 38, 48 and 49 to the PDZ1 domain of PSD-95 protein was demonstrated by NMR spectrometry [49]. The compound number 50 was predicted initially by docking studies and subsequently the prediction was confirmed by biochemical studies [50]. Out of the 13 molecules in the second cluster (cyan colored), K_i_ or K_d_ values were not known for two molecules *i.e.* 56 and 58. Compound number 56 is shown to inhibit PDZ1 domain of NHERF1 [51], while the compound number 58 inhibits PDZ3 domain of PSD-95 protein [52]. Remaining 11 molecules with known K_i_ values in cluster two, have been experimentally characterized to inhibit Dishevelled PDZ domain [24], [53], [54]. Third cluster (pink colored) consists of 39 molecules, which are peptidomimetics where modifications have been introduced at either N or C-terminal of 4-mer or 5-mer peptides. The binding affinity was not known for compound number 59, which is a β strand peptidomimetic and is known to inhibit PDZ domain of α-1 syntrophin [24]. The other 38 molecules in this cluster are N-alkylated tetrapeptides, which inhibit PSD-95/NMDA receptor interaction and have functional implications for compound stability in blood plasma and cell membrane permeability [29]. Moreover, comparative studies regarding binding of these 38 molecules with 2 different PDZ domains of the same protein have also been reported [29]. Since binding affinities were known for these 38 molecules from the third cluster for two different PDZ domains of the same protein, they were chosen for structural analysis of their binding modes and analysis of their selectivity for these two PDZ domains.

### Prediction of Binding Sites and Binding Modes by Docking Studies

**Figure 3:**
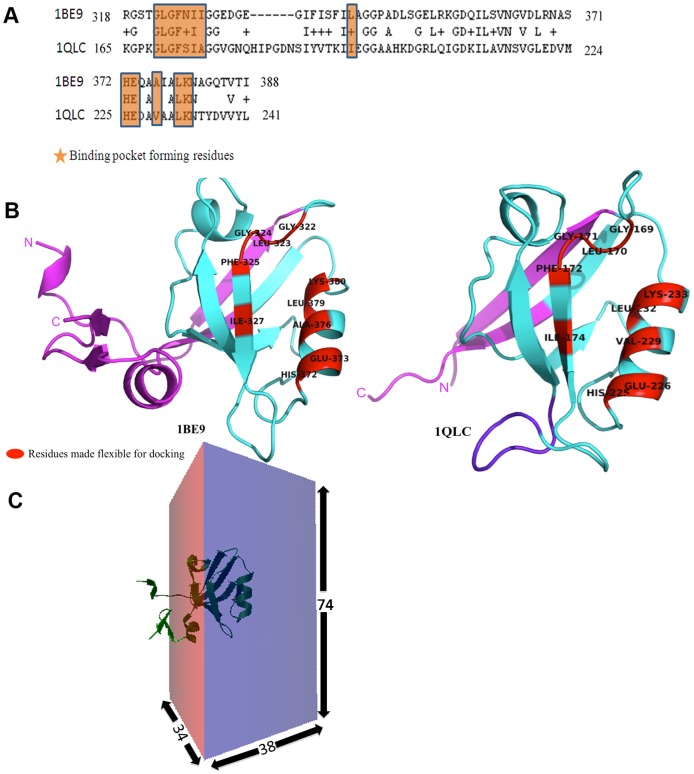
Differences between PDZ2 and PDZ3 of PSD-95. (A) Local alignment of the sequences of 2^nd^ (PDB ID: 1QLC) and 3^rd^ (PDB ID: 1BE9) PDZ domains of PSD-95 protein by BLAST and the conservation of the binding pocket residues is highlighted. (B) Ribbon diagrams depicting the structures 1BE9 and 1QLC. Regions in both 1BE9 and 1QLC, which show alignment in Figure 3A, are colored in cyan, while the N- and C-terminus residues, which did not align, are depicted in magenta. The longer loop region in 1QLC corresponding to the insertion seen in the local alignment is colored in purple. The binding pocket residues made flexible during docking are shown in red color. (C) Schematic depiction of the grid box used for docking of ligands on both PDZ domains. The dimension are x = 38Å, y = 74Å & z = 34Å.

**Figure 4:**
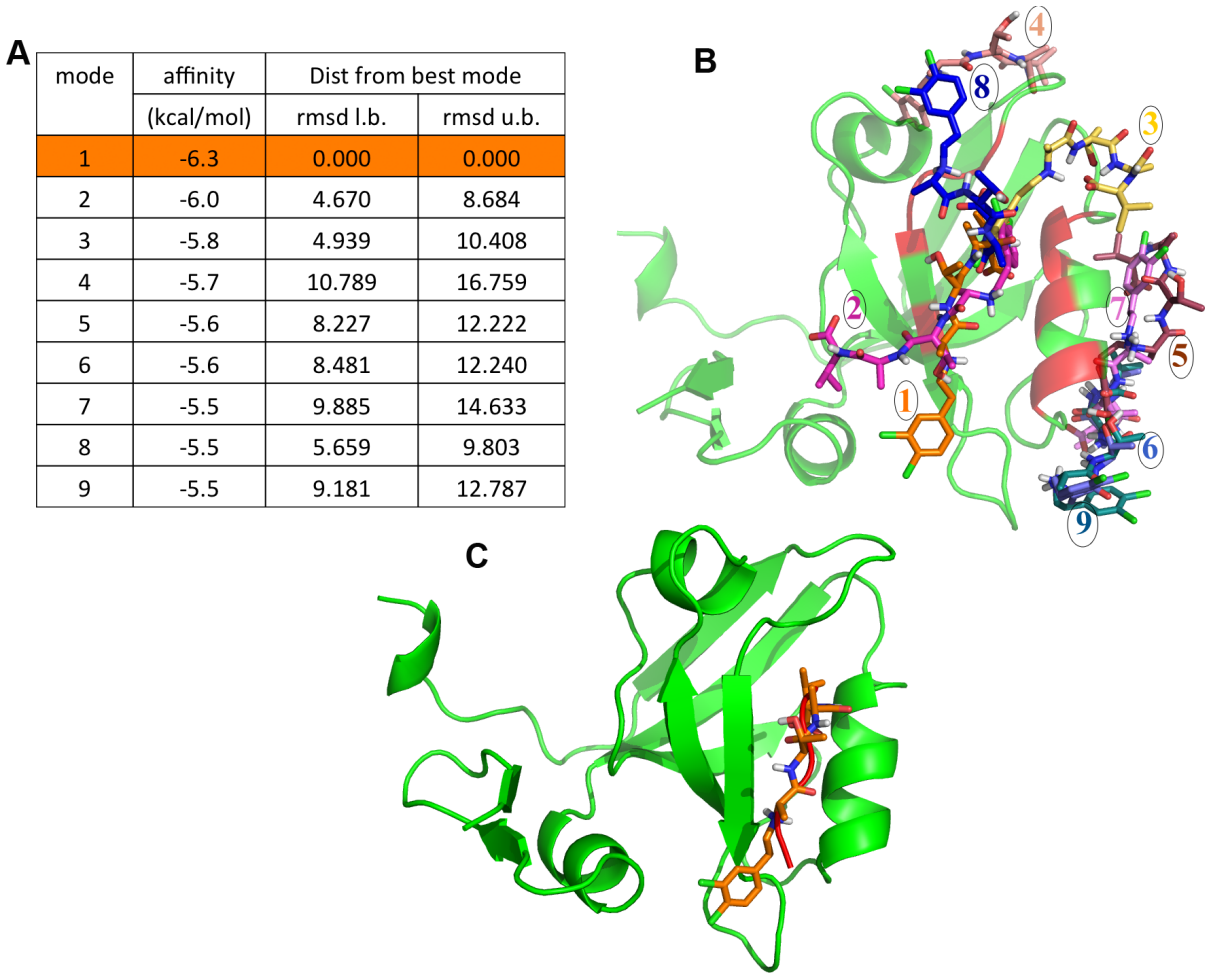
Docking output of 3,4-dichlorophenylethylATAV onto the PDZ3 domain. (A) Typical example of the output of docking done by VINA showing 9 top scoring docking solutions. The lower and upper bound RMSDs for the 9 binding poses have been calculated with respect to the conformation highlighted in orange. (B) Image showing all 9 docked conformations of one of the ligands (3,4-dichlorophenylethylATAV) under study on 1BE9. (C) 1BE9 with its native peptide (red colored) in cartoon representation. The docked conformation of ligand chosen for MD simulation is shown in stick representation.

Before carrying out docking studies for identifying the preferred binding sites of these 38 known inhibitors on second and third PDZ domains of PSD95, we analyzed crystal structures of PDZ domains in complex with the inhibitors to check whether these small molecule inhibitors occupy the same binding pocket as the native peptide substrates. **Figure S3** shows the superposition of the crystal structures of PDZ domain of Shank protein in complex with the peptide (PDB ID: 1Q3P) and also the crystal structure of the same PDZ domain in complex with the inhibitor tetrahydroquinoline carboxylate (PDB ID: 3O5N). As is evident, the small molecule and peptide occupy the same binding pocket. The molecule is mostly interacting with the hydrophobic pocket formed by the GLGF loop and the helix. In the native peptide complex the C-terminal hydrophobic residue of the substrate peptide occupies this hydrophobic pocket. In order to understand the structural basis of differential selectivity of PDZ2 and PDZ3 domains of PSD-95 for the same inhibitors, the sequence and structural differences between these two PDZ domains were also compared. **Figure 3A** shows the BLAST alignment between the sequences of 2^nd^ (PDB ID: 1QLC) and 3^rd^ (PDB ID: 1BE9) PDZ domain of PSD-95 protein, while **Figure 3B** shows the corresponding structures in the cartoon representation. As can be seen, the local alignment of the sequences of these two PDZ domains cover 77 residues which encompass the core of the PDZ domains, while they do not show sequence similarity in their N- and C-terminal stretches. In fact N- and C-terminal regions of the PDZ3 that do not align with the corresponding regions of PDZ2 are longer in size and PDZ3 contains the extra domain helix in the C-terminal stretch. Apart from these differences in the N- and C-terminal sequence stretches, the core region of these two domains show an identity of 40% and similarity of 56% over 77 residues. In fact the binding pocket residues are almost identical in these two domains except for few conservative substitutions involving N326S, L342I and A376V. However, the crucial difference between these two PDZ domains in the core region is the six-residue insertion in PDZ2 with respect to PDZ3 in the loop joining β strands βB and βC. Thus, between these two PDZ domains the longer loop between β strands βB and βC in PDZ2 and the C-terminal extra domain helix in the PDZ3 are the crucial structural differences which might affect the inhibitor binding and hence give rise to differential selectivity. All the 38 inhibitors in cluster 3 were docked onto 1QLC and 1BE9 for elucidating how these structural differences affect the binding modes. **Figure 3C** shows the docking grid around the peptide-binding site in these PDZ domains and it covers the most of the PDZ core. The specificity determining residues (SDR) in the binding pockets (shown in RED color in **Figure 3B**) of these two PDZ domains were made flexible during the docking. Batch docking runs were carried out for 1QLC and 1BE9 using AUTODOCK VINA software. It may be noted that the bound peptide was removed from 1BE9 before docking. **Figure 4** shows the final results obtained from a typical VINA docking run using docking of the inhibitor 3,4-dichlorophenylethylATAV onto the PDZ3 domain, 1BE9. **Figure 4A** shows the binding energy (VINA docking score) values for 9 top scoring ligand poses and RMSDs between their heavy atoms. **Figure 4B** shows these 9 bound conformations that have been explored by the ligand on the PDZ domain. As can be seen, only two out of these nine conformations bind at the same site as the native peptide on the PDZ domain. **Figure 4C** shows the docking pose that has minimum RMSD from the native peptide and in this particular case this happens to be the conformation having the highest binding affinity or VINA docking score. However, detailed analysis of the docking results for all the 38 ligands on two PDZ domains *i.e.* PDZ2 (1QLC) and PDZ3 (1BE9) revealed that quite often the highest affinity binding pose was away from the peptide binding site. Therefore, the final docking pose from each run was selected by visual inspection using the criteria that the ligand should be in a conformation parallel to the βB strand of the PDZ domain as depicted in **Figure 4C** and the carboxyl terminal of the peptidomimetic ligands should lie in the hydrophobic pocket made by GLGF motif of the PDZ domain. Thus, based on the docking results most appropriate binding poses were identified for each of these 38 peptidomimetic ligands on PDZ2 and PDZ3 domains of PSD-95.

### Refinement of Docked PDZ-inhibitor Complexes by Explicit Solvent MD Simulations

**Figure 5:**
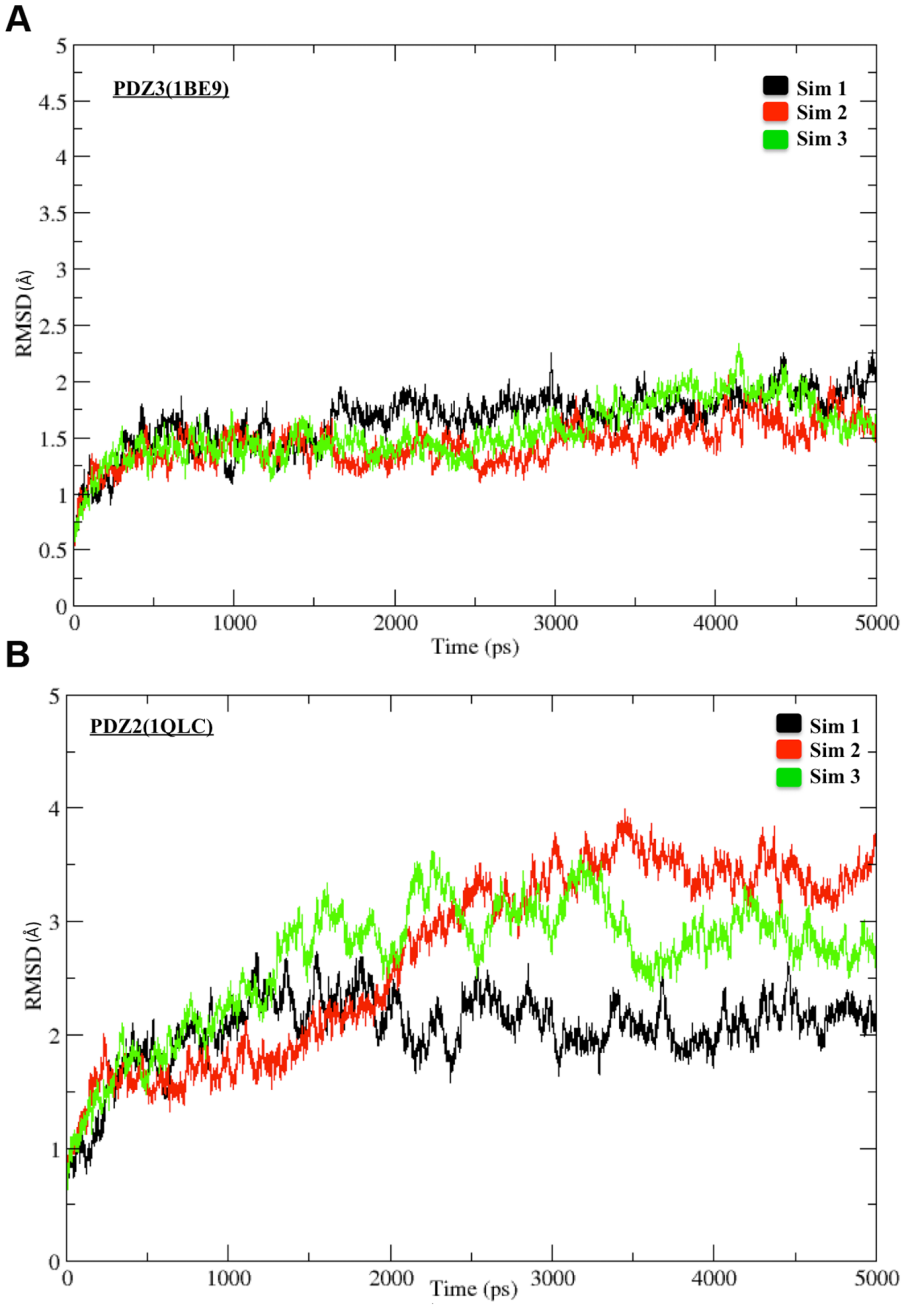
RMSD *vs* Time plot. (A) RMSD *vs* time plot for the MD simulation on the docking complex involving the inhibitor 3,4-dichlorophenylethylATAV and 1BE9. (B) RMSD *vs* time plot for the MD simulation on the docking complex involving the inhibitor 3,4-dichlorophenylethylATAV and 1QLC.

**Figure 6:**
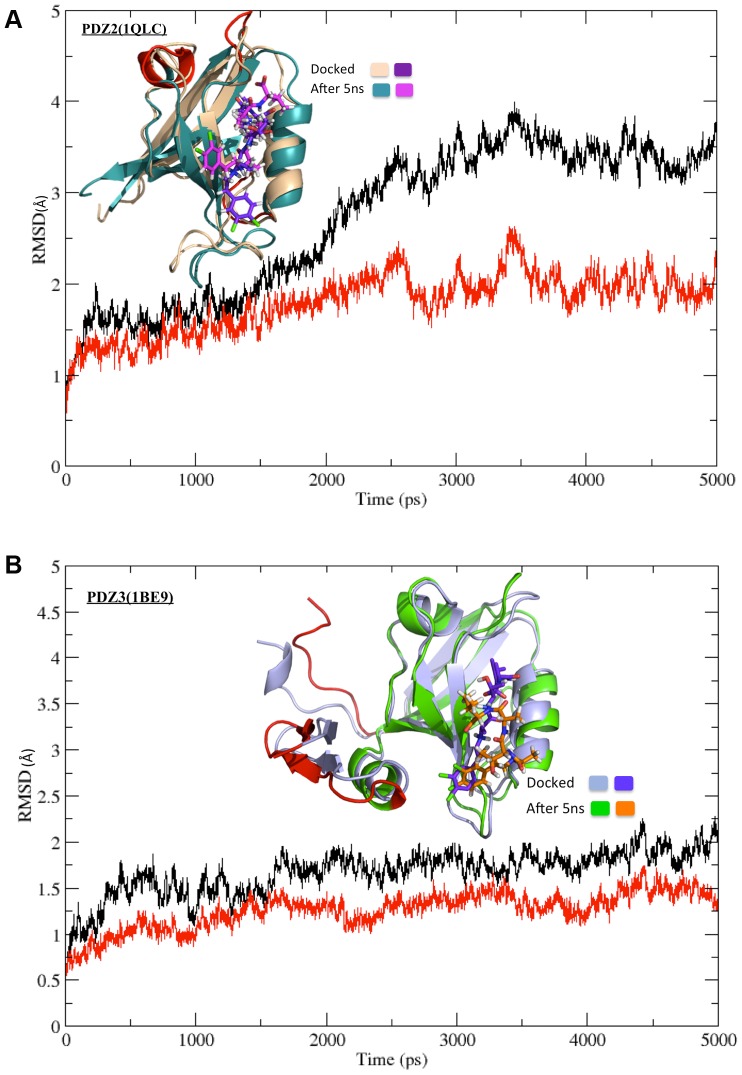
RMSD *vs* Time plot after excluding certain residues. (A) RMSD *vs* time plot for one of the MD simulations (simulation 2 from **Figure 5B**) on the docking complex involving the inhibitor 3,4-dichlorophenylethylATAV and 1QLC. RMSD has been calculated after excluding residues 12–15 from N-terminal and loop regions comprising of residues 42–49 & 65–70. The inset picture shows the superposition of the docked complex before and after MD. (B) RMSD *vs* time plot for one of the MD simulations (simulation 1 from **Figure 5A**) on the docking complex involving the inhibitor 3,4-dichlorophenylethylATAV and 1BE9. RMSD has been calculated after excluding residues 1–10 from N-terminal and 100–115 from C-terminal of the protein. The inset picture shows the superposition of the docked complex before and after MD.

**Figure 7:**
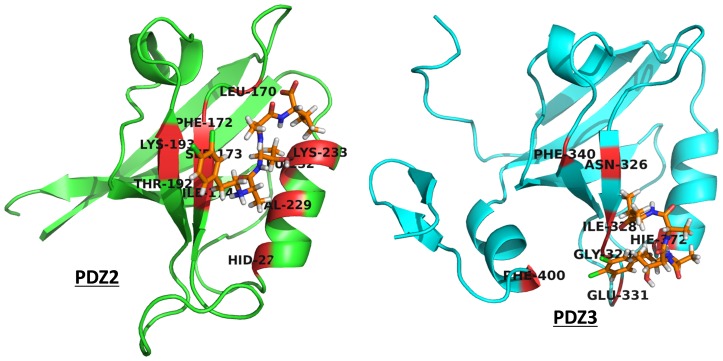
Binding modes of ligands. The binding modes of the ligand 3,4-dichlorophenylethylATAV to the 2^nd^ and 3^rd^ PDZ domains of PSD95 as predicted by docking and MD simulations. The residues of the PDZ domains, which are in contact with the inhibitor for more than 50% of the simulation time during last 1ns of the 5ns MD simulations, are shown in RED color.

The docking study carried out to obtain the structures of PDZ-inhibitor complexes permitted flexibility of only a limited number of binding pocket residues which have been identified from analysis of PDZ-peptide complexes. The peptidomimetic inhibitors contain additional functional groups, which made contact with other regions in the binding pocket of the PDZ domains that were rigid during the docking calculations. Therefore, it was necessary to investigate the role of receptor flexibility on the binding of these inhibitors. Hence, the final docking poses for each of these compounds on PDZ2 and PDZ3 domains of PSD-95 were further refined by explicit solvent MD simulations. In order to enhance the conformational sampling, simulations on each PDZ-inhibitor complex was also repeated starting from the same initial structure, but assigning different initial velocities by changing the random number seed. **Table 1** provides the serial number and name of the inhibitor, name of the receptor PDZ domain, length and number of repeat runs for each simulation. As can be seen, a total of 228 explicit solvent MD simulations of 5 ns duration was carried out to refine the PDZ-inhibitor complexes obtained from docking. In order to monitor the structural changes in the PDZ-inhibitor complexes during the simulation, root-mean-square deviation (RMSD) for the backbone atoms of the PDZ and the inhibitor with respect to the initial structure was calculated for each MD trajectory. **Figure 5A** shows as a representative example, the RMSD *vs* time plot for the complex involving the ligand 3,4-dichlorophenylethylATAV and PDZ3 domain of PSD-95, while **Figure 5B** shows the RMSD *vs* time plot for the same ligand in complex with PDZ2 domain. As can be seen from **Figure 5A** for the complex involving PDZ3 domain, the RMSD values in all 3 simulations remain lower than 2Å throughout the 5 ns trajectory, thus suggesting that even after introducing receptor and ligand flexibility there are no large conformational changes in the complex and the inhibitor remains in the peptide binding pocket. On the other hand, in **Figure 5B** the RMSD values in two out of the three simulations increase to 3.5Å, thus indicating significant conformational changes in the complex. Detailed analysis of the RMSF values for the various residues in different trajectories indicated that the larger overall RMSD in case of PDZ2 domain arises from the small helical stretch connecting βC to βD, and the loop regions connecting βD to αB and βA to βB. However, the inhibitor 3,4-dichlorophenylethylATAV remains bound in the peptide-binding pocket similar to the complex involving PDZ3 domain. **Figure 6A** shows the RMSD *vs* time plot excluding these regions showing higher flexibility in simulation number 2 for the complex between 3,4-dichlorophenylethylATAV and PDZ2. Inset in the **Figure 6A** shows the superposition of the final structure obtained after simulation of the complex on the initial structure obtained from docking. As can be seen, the inhibitor remains close to the site identified by docking and the larger RMSD arises from structural changes in the loop regions shown in red color. **Figure 6B** shows the RMSD *vs* time plot for the complex between 3,4-dichlorophenylethylATAV and PDZ3 excluding the terminal regions and inset shows the superposition of the final structure obtained after simulation of the complex on the initial structure obtained from docking. It was interesting to find out the residues from both the PDZ domains with which these N-alkylated tetrapeptides interact. For each PDZ-inhibitor complex, interacting residues of both PDZ2 and PDZ3 domains were identified following the procedure described in the methods section. For identifying the interacting residues after simulation, snapshots were taken at an interval of 50 ps from the last 1 ns of the trajectory and percentage occupancy of the contacts were calculated, and only those contacts for which occupancy was more than 50% were considered. **Figure 7** shows the interacting residues from both PDZ2 and PDZ3 domains with the ligand 3,4-dichlorophenylethyATAV after MD simulations. It is indeed interesting that despite having longer terminal stretches PDZ3 shows smaller fluctuations in these terminal regions compared to PDZ2, because of the stable interactions between extra domain helix and PDZ core region.

**Table 1:** Table summarizing the list of inhibitors for which MD simulations performed in complex with PDZ2 (1QLC) and PZD3 (1BE9).

S. No.	Ligand	PDZ2 (1QLC)	PDZ3 (1BE9)
**1**	AdamantylethylETAV	5 ns (3)	5 ns (3)
**2**	BicycloheptylmethylETAV	5 ns (3)	5 ns (3)
**3**	BicycloheptylethylETAV	5 ns (3)	5 ns (3)
**4**	3,4dichlorophenylethylATAV	5 ns (3)	5 ns (3)
**5**	3,4dichlorophenylethylETAsV	5 ns (3)	5 ns (3)
**6**	3,4dichlorophenylethylETAV	–	5 ns (3)
**7**	3,4dichlorophenylethylETDV	5 ns (3)	5 ns (3)
**8**	AnthracenethylETAV	5 ns (3)	5 ns (3)
**9**	AnthracenpropylETAV	5 ns (3)	5 ns (3)
**10**	ATAV	5 ns (3)	5 ns (3)
**11**	ATDV	5 ns (3)	5 ns (3)
**12**	CycloheptylmethylETAV	5 ns (3)	5 ns (3)
**13**	CycloheptylethylETAV	5 ns (3)	–
**14**	ETAV	5 ns (3)	5 ns (3)
**15**	ETDV	5 ns (3)	5 ns (3)
**16**	Naphthalene2ylethyETAV	5 ns (3)	5 ns (3)
**17**	Naphthalene2ylethylATAV	5 ns (3)	5 ns (3)
**18**	Naphthalene2ylethylETAsV	5 ns (3)	5 ns (3)
**19**	Naphthalene2ylethylETDV	5 ns (3)	5 ns (3)
**20**	NaphthalenepropylETAV	5 ns (3)	5 ns (3)
**21**	N-cyclohexlyethylETAsV	5 ns (3)	5 ns (3)
**22**	N-cyclohexylethylATAV	5 ns (3)	5 ns (3)
**23**	N-cyclohexylethylATDV	5 ns (3)	5 ns (3)
**24**	N-cyclohexylethylETAV	5 ns (3)	5 ns (3)
**25**	N-cyclohexylethylETDV	5 ns (3)	5 ns (3)
**26**	N-cyclohexylethylQTAV	5 ns (3)	5 ns (3)
**27**	N-cyclohexylethylQTDV	5 ns (3)	–
**28**	NmethylEsTAV	5 ns (3)	5 ns (3)
**29**	NmethylETAsV	5 ns (3)	5 ns (3)
**30**	NmethylETAV	5 ns (3)	5 ns (3)
**31**	NmethylETsAV	5 ns (3)	5 ns (3)
**32**	PyrenbutylETAV	5 ns (3)	5 ns (3)
**33**	PyrenethylETAV	5 ns (3)	5 ns (3)
**34**	QTAV	5 ns (3)	5 ns (3)
**35**	QTDV	5 ns (3)	5 ns (3)
**36**	TrifluorophenylethylETAV	5 ns (3)	–
**37**	KQTSV	5 ns (3)	5 ns (3)
**38**	IESDV	5 ns (3)	5 ns (3)

Second and third column indicate length of each simulation with numbers in the bracket indicating the number of repeats for each MD run.

As can be seen from **Figure 7** in case of PDZ2 domain the ligand 3,4-dichlorophenylethylATAV remains in the peptide-binding pocket at the end of the 5ns MD simulations. However, in case of PDZ3, even though the ligand remains in the peptide-binding pocket of the PDZ domain, the C-terminal of the ligand has moved away from the carboxylate-binding loop. Similar trend was observed in all three simulations. It is interesting to note that, 3,4-dichlorophenylethylATAV has a K_i_ value of 13 µM for PDZ2, while the binding affinity for PDZ3 domain is 64 µM. It is possible that the ligand has deviated significantly from the peptide-binding pocket of PDZ3 during MD simulation because of its lower binding affinity for this domain. We wanted to investigate if similar trend is observed for simulations on other PDZ-inhibitor complexes. The various inhibitors were ranked in terms of their experimental binding affinities for PDZ2 and PDZ3 domains, lowest K_i_ or K_d_ values having rank 1 and highest K_i_ or K_d_ values having rank 38. **Table 2** shows the binding affinity ranks for all the inhibitors and whether they have moved away from the binding pocket during the MD simulations. It was observed that in case of PDZ2 domain, ligands having ranks 32–33 and 36–38 had moved away from the canonical peptide binding site during the MD simulations (highlighted in red color in **Table 2**). The corresponding K_i_ values ranged from 33 µM to 220 µM. On the other hand, all ligands having K_i_ values lower than 33µM for PDZ2 domain remained in the peptide binding pocket. In case of PDZ3 the ligands corresponding to ranks 29–38 for which the experimental binding affinity were not measurable as they were nonbinders, 8 out of these 10 ligands drifted away from the peptide binding pocket of PDZ3 domain. Similarly out of the 12 other low affinity ligands for PDZ3 domain which had K_i_ values higher than 64 µM, 8 ligands drifted away from the binding pocket. Thus, our results indicate that, deviation of the bound ligand from the peptide binding pocket during MD simulations can be used as an indicator for the low binding affinity of the ligand. Since many of the ligands in our dataset had lower affinity for the PDZ3 domain compared to PDZ2 domain, in case of PDZ3 domain more ligands drifted away from the peptide binding pocket. Thus incorporation of ligand and receptor flexibility by MD simulation has helped in qualitatively rationalizing the experimentally observed differential selectivity of the ligands for PDZ domains. Our analysis also revealed that, the differential selectivity primarily arises from the differences in the conformation of the loop connecting the βB and βC strands.

**Table 2:** Table summarizing the ranking of the inhibitors according to their K_i_ values.

S. No.	Ligand	Rank (PDZ2) (1QLC)	Rank (PDZ3) (1BE9)
**1**	AdamantylethylETAV	15	3
**2**	BicycloheptylmethylETAV	10	14
**3**	BicycloheptylethylETAV	12	12
**4**	3,4dichlorophenylethylATAV	26	**17**
**5**	3,4dichlorophenylethylETAsV	5	5
**6**	3,4dichlorophenylethylETAV	7	4
**7**	3,4dichlorophenylethylETDV	9	**21**
**8**	AnthracenethylETAV	23	2
**9**	AnthracenpropylETAV	21	20
**10**	ATAV	**36**	26
**11**	ATDV	**33**	**29**
**12**	CycloheptylmethylETAV	11	13
**13**	CycloheptylethylETAV	8	7
**14**	ETAV	29	**24**
**15**	ETDV	28	**30**
**16**	Naphthalene2ylethyETAV	4	8
**17**	Naphthalene2ylethylATAV	34	25
**18**	Naphthalene2ylethylETAsV	6	10
**19**	Naphthalene2ylethylETDV	14	**23**
**20**	NaphthalenepropylETAV	19	16
**21**	N-cyclohexlyethylETAsV	1	9
**22**	N-cyclohexylethylATAV	20	**19**
**23**	N-cyclohexylethylATDV	16	**27**
**24**	N-cyclohexylethylETAV	2	11
**25**	N-cyclohexylethylETDV	3	31
**26**	N-cyclohexylethylQTAV	25	**22**
**27**	N-cyclohexylethylQTDV	18	**32**
**28**	NmethylEsTAV	31	**33**
**29**	NmethylETAsV	24	**34**
**30**	NmethylETAV	22	35
**31**	NmethylETsAV	**38**	**36**
**32**	PyrenbutylETAV	**32**	18
**33**	PyrenethylETAV	27	15
**34**	QTAV	**37**	**28**
**35**	QTDV	35	**37**
**36**	TrifluorophenylethylETAV	13	6
**37**	KQTSV	30	1
**38**	IESDV	17	**38**

Second and third column indicate the rank of the inhibitors according to their K_i_ vales determined for PDZ2 and PDZ3 domains of PSD-95 protein (lowest K_i_- Rank1; highest K_i_ – Rank38).

Numbers in BOLD indicate inhibitors which are drifting away from the binding pocket.

### Calculation of Binding Free Energy Values for Various PDZ-inhibitor Complexes

In order to make a quantitative comparison with the experiments, binding free energy values were also calculated from MD trajectories using MM/PBSA for all 38 PDZ-inhibitor complexes. **Table S1** shows for each of the 38 inhibitor-PDZ pairs corresponding to the 2^nd^ PDZ domain of PSD-95, the experimental binding free energy values calculated from reported K_d_ or K_i_ values, the binding affinities calculated by VINA and MM/PBSA binding energy values computed from explicit solvent MD trajectories. Docking of the inhibitor 3,4-dichlorophenylethylETAV (inhibitor 6) on PDZ2 did not give any solution in the peptide binding site, therefore no MD simulation has been carried out on this complex and **Table S1** does not give computed binding energy values for this complex. However, experimental studies suggest that this molecule binds to PDZ2 with high affinity. Experimental data indicate that both the peptides with no modifications (37 & 38 in **Table S1**) bind to PDZ2, but IESDV binds with a higher affinity compared to KQTSV [27], [37]. Interestingly, the VINA docking score as well as the MM/PBSA binding energy calculated from the docked complex also show the same trend. However, MM/PBSA binding energy calculated after MD simulations shows a much higher difference in the binding energy values for these two peptides, though the trend is similar to the experimental binding energy values. It is known from previous studies that Val at position 0 (1^st^ position from C-terminal) and Ser or Thr at position −2 (3^rd^ position from C-terminal) of the substrate peptide are the preferred residues and they play a deterministic role in defining the affinity [13]. Thus, our simulations have reproduced these experimental observations. The compounds 10, 11, 14, 15, 34 and 35 in **Table S1** are also peptides without modifications. Comparison of the experimental binding affinities of these six peptides indicate that Glu at position −3 is important for affinity whereas Asp at position −1 is dispensable *i.e.* it can be replaced by Ala or Gln without significant loss in affinity (34 & 35 in **Table S1**). It is encouraging to note that the computed binding energy values also qualitatively reproduce this observed differential binding affinities for different peptides with substitutions at these specificity-determining positions on the substrate. However, for some of these peptides the computed energy differences do not correlate with experimental affinities. For example, in case of compounds 10 and 11, ATDV shows more favorable binding energy compared to ATAV in terms of VINA score as well as MM/PBSA energy computed from docked complexes in agreement with experimental results. However, MM/PBSA binding energy calculated from MD simulations shows a reverse trend for ATAV *vs* ATDV, which is contrary to the experimental observation. It is seen that substitution of Asp at 2^nd^ position with Ala reduces affinity in case of 14 and 15, and interestingly the MM/PBSA binding energies calculated after MD simulations are also in agreement with experiments. Similarly, the experimental binding affinity differences between the ETDV and N-cyclohexylethyl-ETDV (15 &25 in **Table S1**) could also be qualitatively explained by our MM/PBSA analysis. **Table S2** shows the experimental binding free energy for the 38 inhibitors for the PDZ3 domain and their comparison with binding energies computed by AUTODOCK VINA, and MM/PBSA analysis before and after MD simulations. As can be seen, PDZ3 possesses no affinity towards IESDV peptide (38 in **Table S2**), but it binds to KQTSV (37 in **Table S2**). This was also seen in terms of binding affinity calculated from docking studies and binding energy values calculated after MD simulations on selected docked poses. In cases where the experimentally determined K_i_ values are very high or the compounds show no affinity towards PDZ3, similar results were reflected in docking studies as well as in MD simulations.

**Figure 8:**
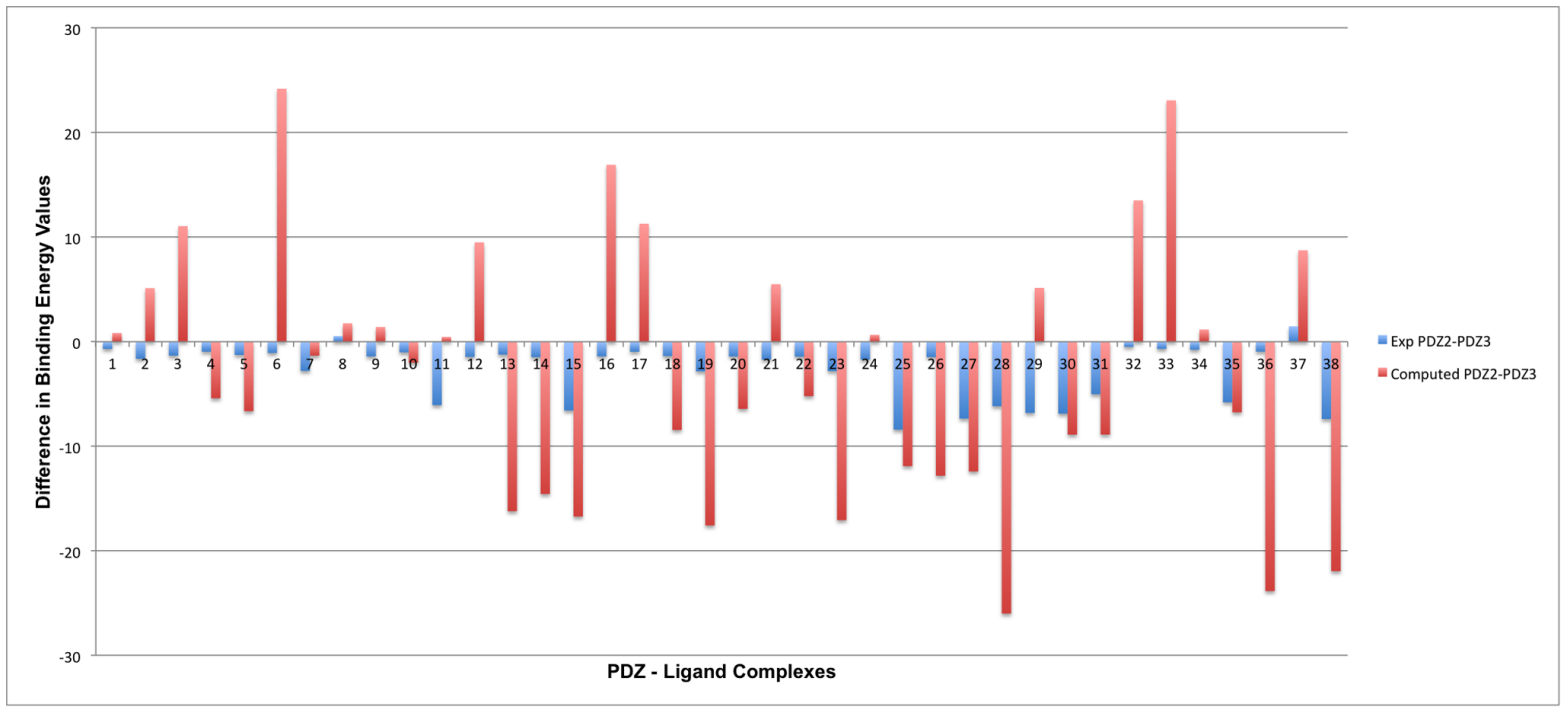
Comparative analysis for determining binding selectivity of PDZ2 and PDZ3. Bar graph showing the comparison of binding energy values determined experimentally as well as computed by using MM/PBSA after 5 ns of MD simulation for the set of 38 peptidomimetic ligands for both PDZ2 and PDZ3 domains. The ligand numbers given on x-axis is according to the numbering given in **Table 1**. The difference in binding energy values between PDZ2 and PDZ3 for all 38 ligands is plotted on y-axis.

In order to understand the differential specificity of these 38 inhibitors towards PDZ2 and PDZ3 domains, a comparative analysis of the binding affinities determined experimentally as well as computed by MM/PBSA after MD simulations was done. **Figure S4A** and **S4B** show the MM/PBSA and experimental binding free energy values for all 38 ligands, where they have been divided into two groups depending upon the type of N-terminal modifications done on the tetrapeptide backbone. As seen in **Figure S4A**, in terms of experimental binding energies, the compounds 4 and 5 show higher binding affinity towards PDZ2 compared to PDZ3 domain. The same trend is also reproduced by MM/PBSA calculations. Similarly, as can be seen from **Figure S4B** in experimental studies the compounds 28 and 31 bind to PDZ2 domain only. Accordingly, the MM/PBSA energy values for these two compounds for PDZ2 domain is much more negative compared to PDZ3 domain. Based on similar analysis for all 38 ligands, **Figure 8** shows the difference in experimental as well as computational binding energy values for PDZ2 and PDZ3 domains. It is interesting to note that, even though the magnitude of the binding energy differences obtained from simulation and experiments differ, the trend is similar in 23 out of 38 cases. In five other cases, where ligands show very similar binding affinity for both the PDZ domains, the differences in MM/PBSA binding energy values are in the range 0.84 to 1.41 kcal/mole, while the corresponding differences in experimental binding energy values range from −0.69 to −1.70 kcal/mole. Thus in 28 out of 38 cases differential binding affinities of ligands for PDZ2 and PDZ3 domains predicted by MM/PBSA calculations are in qualitative agreement with experimental results. Only in 10 cases, the trend in binding energy differences for PDZ2 and PDZ3 domains obtained from MM/PBSA calculations differ from those obtained from experimental binding energy values. Thus our results suggest that, the simulations are able to predict the differential selectivity of ligands by different PDZ domains in majority of the cases.

### Correlation between Computed and Experimental Binding Free Energies

**Figure 9:**
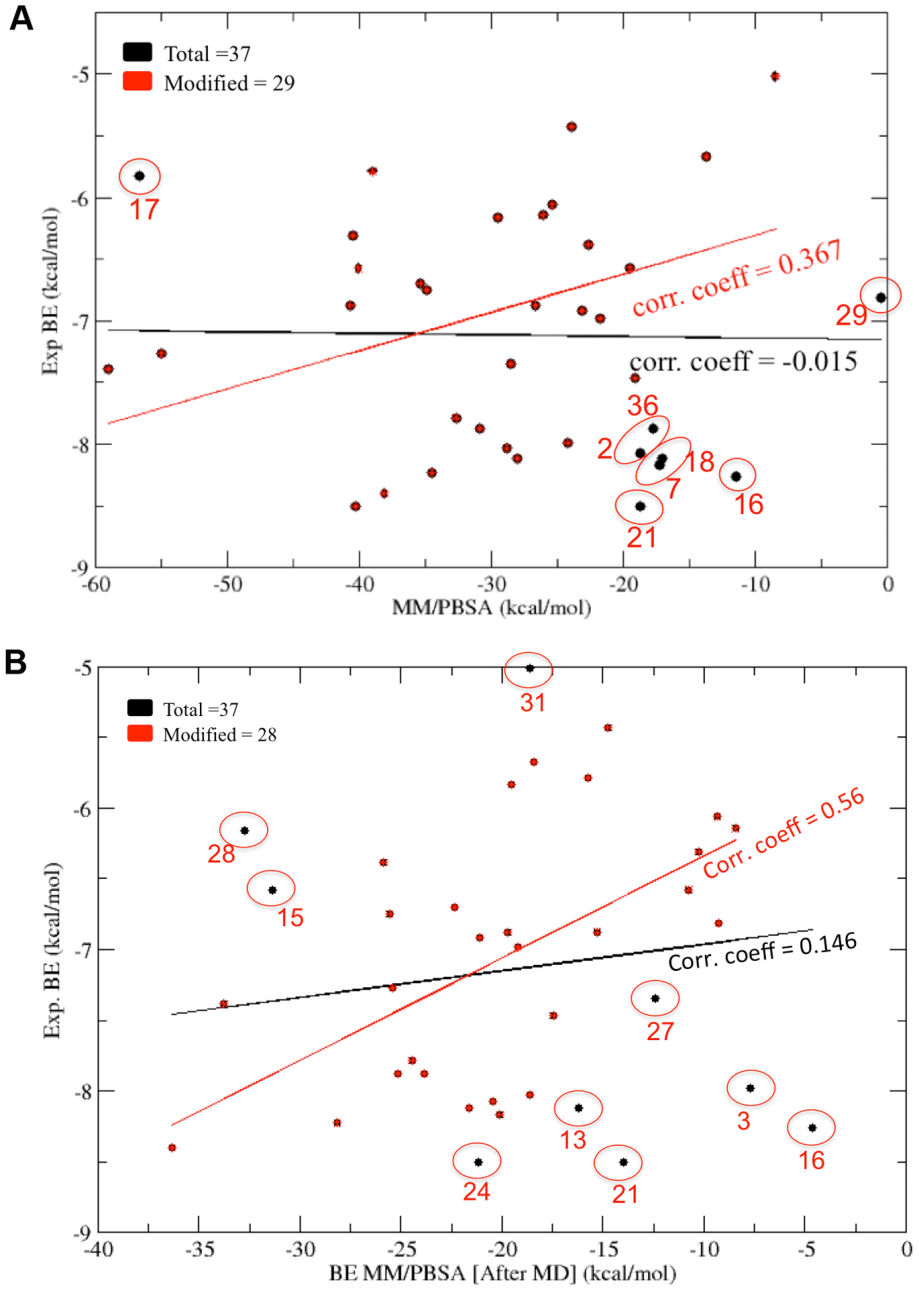
Correlation with experiments (PDZ2). (A) Correlation between experimentally determined binding energy values and binding energy values calculated from MM/PBSA for the energy minimized docked complexes for 2^nd^ PDZ domain of PSD-95 protein. (B) Correlation between experimentally determined binding energy values and binding energy values calculated from MM/PBSA after performing MD simulation on the docked complexes for 2^nd^ PDZ domain of PSD-95 protein.

**Figure 10:**
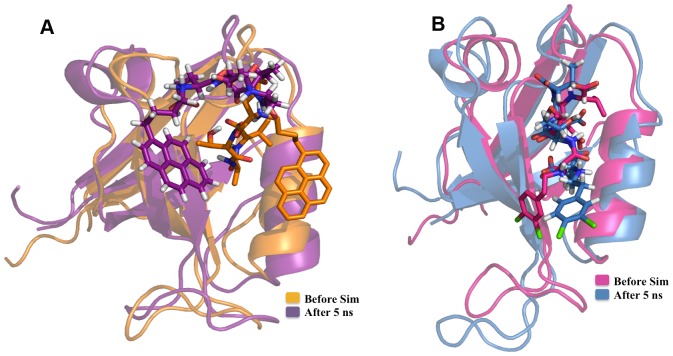
Comparison before and after MD (PDZ2). (A) Structural superimposition of docked complex of one of the ligands (PyrenbutylETAV) before and after MD simulation on 2^nd^ PDZ domain of PSD-95 protein (PDBID: 1QLC) where the ligand is moving out of the binding pocket of 1QLC during the course of simulation. (B) Structural superimposition of docked complex of one of the ligands (3,4-dichlorophenylethylETDV) before and after MD simulation on 2^nd^ PDZ domain of PSD-95 protein (PDBID: 1QLC) where the ligand is repositioning itself in the binding pocket of 1QLC during the course of simulation.

**Figure 11:**
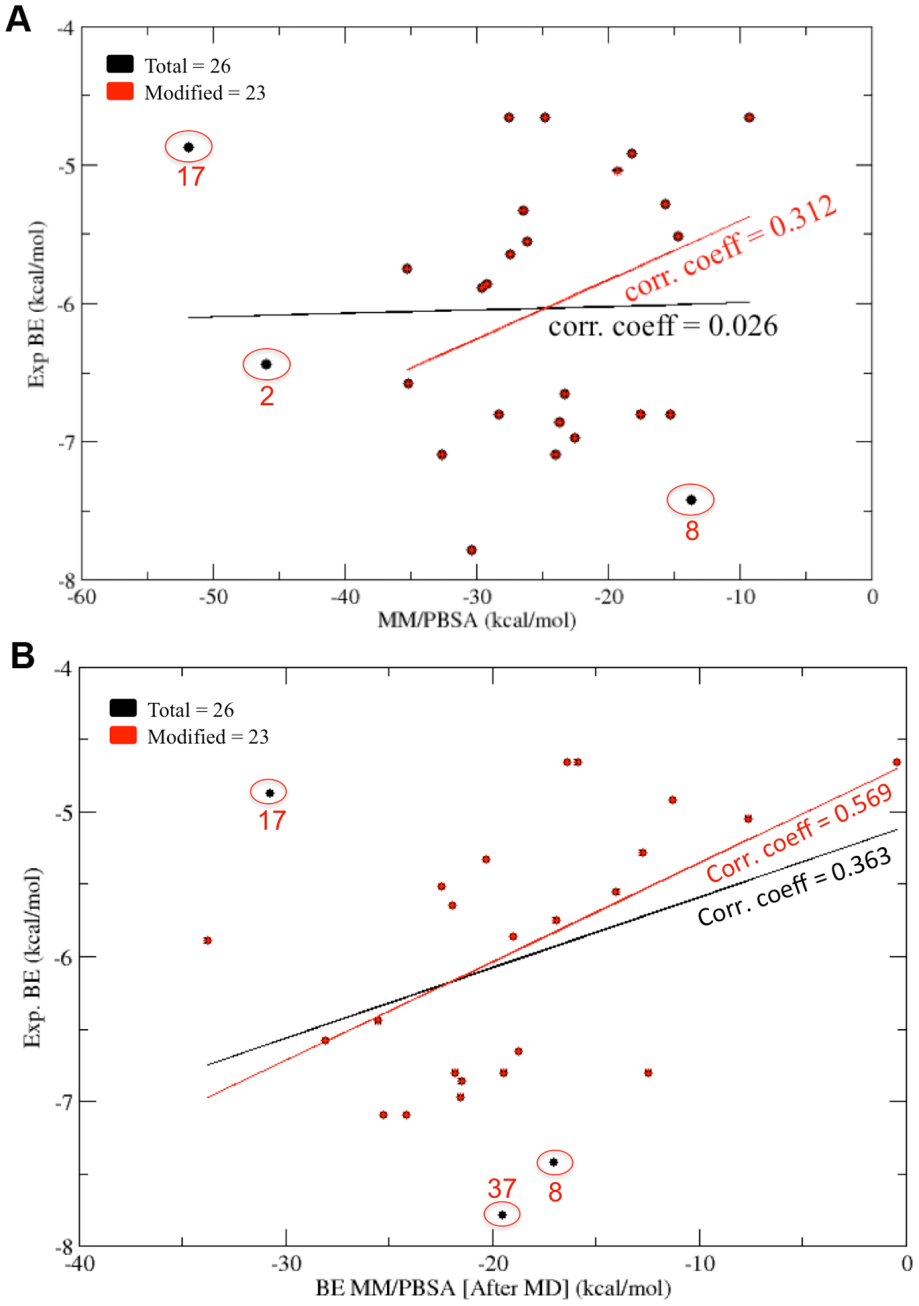
Correlation with experiments (PDZ3). (A) Correlation between experimentally determined binding energy values and binding energy values calculated from MM/PBSA for the minimized docked complexes for 3^rd^ PDZ domain of PSD-95 protein. (B) Correlation between experimentally determined binding energy values and binding energy values calculated from MM/PBSA after performing MD simulation on the docked complexes for 3^rd^ PDZ domain of PSD-95 protein.

**Figure 12:**
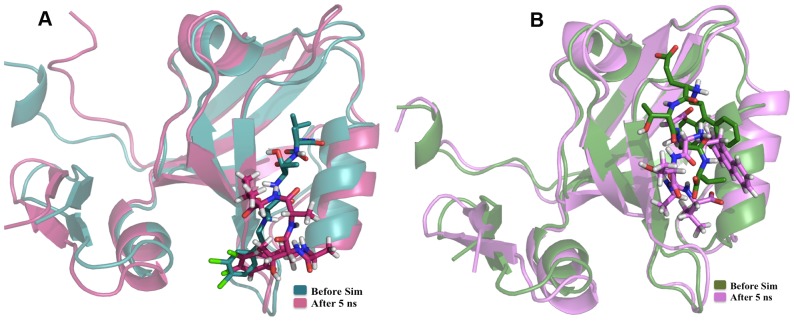
Comparison before and after MD (PDZ3). (**A**) Structural superimposition of docked complex of one of the ligands (3,4-dichlorophenylethylATAV) before and after MD simulation on 3^rd^ PDZ domain of PSD-95 protein (PDBID: 1BE9) where the ligand is moving out of the binding pocket of the 1BE9 during the course of simulation. (B) Structural superimposition of docked complex of one of the ligands (AnthracenethylETAV) before and after MD simulation on 3^rd^ PDZ domain of PSD-95 protein (PDBID: 1BE9) where the ligand is repositioning itself in the binding pocket of the 1BE9 during the course of simulation.

The MM/PBSA binding energy values calculated before and after MD simulations were compared with the experimental binding energy values for both PDZ2 and PDZ3 domains. **Figure 9A** shows the correlation between MM/PBSA binding energy values calculated before MD simulations and experimental binding energy values for PDZ2. As can be seen, the correlation coefficient for all 37 inhibitors is −0.015 and even after removing eight compounds, the correlation coefficient increases to 0.367 only. Interestingly, the MM/PBSA binding energy values calculated after MD simulations showed better correlation with experimental binding energies with a correlation coefficient of 0.56 after removing outliers (**Figure 9B**). From these results it is apparent that when both receptor and ligand flexibilities have been introduced, the computed binding energy values show better correlation with experimental results. **Figure 10A** shows the superposition of the PDZ2-inhibitor complex involving the molecule PyrenbutylETAV before and after MD simulations. As can be seen, the ligand is moving out of the binding pocket during MD simulations. On the other hand in case of the complex involving 3,4-dichlorophenylethylETDV and PDZ2 (**Figure 10B**) during the simulation the ligand rearranges its conformation and orientation inside the peptide-binding pocket. These results indicate that ligand and receptor flexibilities incorporated by MD simulations helps in improving correlations between experiment and simulations. The correlation between experimental and computed binding energy values for PDZ3-inhibitor complexes was also found to be similar to that of PDZ2-inhibitor complexes. After removing the outliers, MM/PBSA energies before MD simulations gave a correlation coefficient of 0.312 only (**Figure 11A**). However, as can be seen from **Figure 11B**, the MM/PBSA binding free energy values calculated after MD simulations show a correlation coefficient of 0.363 with experimental binding energies if all the 26 complexes are considered and it increases to 0.569 if three outliers are excluded. These results also reiterate the role of ligand and receptor flexibility in modeling of PDZ-inhibitor complexes and better correlation with experimental binding energy values was obtained because of conformational rearrangement of ligands in the binding site. **Figures 12A and 12B** show typical changes in the binding poses for the inhibitors 3,4-dichlorophenylethylATAV and AnthracenethylETAV, which are facilitated by MD simulations and help in improving correlation with experimental results. We also analyzed the correlation between VINA docking score and experimental binding energy values (**Figure S5**). As can be seen, for PDZ2 the correlation coefficient is 0.3 if all 37 inhibitors are considered and it increases to 0.6 when five outliers are excluded (**Figure S5A**). Similarly in case of PDZ3 domain, after removing the outliers the VINA score showed a correlation coefficient of 0.543 with experimental binding energies (**Figure S5B**). It was interesting to note that, correlation between VINA score and experimental binding energy was comparable to correlations obtained for MM/PBSA binding energy after MD simulations. However, it was surprising that MM/PBSA binding energy before MD simulation shows poor correlation with experimental binding energy compared to VINA score, even though very similar inhibitor bound complexes have been used in both calculations. This apparent discrepancy might be attributed to differences in the VINA scoring function and MM/PBSA forcefield. Generally scoring functions used in docking studies have been parameterized keeping in mind limited flexibilities of receptor-ligand complexes, while MM/PBSA energy functions have been designed for all atom simulations which involve complete flexibilities. Therefore, only after inclusion of flexibilities by MD simulations, MM/PBSA binding energies show similar correlation as VINA docking scores.

## Discussion

Analysis of the structural diversity of the various experimentally characterized inhibitors of PDZ-peptide interactions revealed that they can be divided into three distinct groups based on similarities in their chemical structures. A set of 38 N-alkylated tetrapeptides belonging to one of these three groups were considered for detailed structural analysis of their binding modes as experimental binding affinities for these 38 peptidomimetics for PDZ2 as well as PDZ3 domains of PSD-95 protein were known. Binding modes for these 38 inhibitors on PDZ2 and PDZ3 domains were identified by using a combination of docking and explicit solvent MD simulations, which take into account complete flexibilities of the ligand as well as the receptor. Based on docking studies using AUTODOCK VINA, a set of 9 high scoring docking poses were identified for each inhibitor and the conformation which satisfies the criteria, that carboxyl terminal of the peptidomimetic lies in the hydrophobic pocket of the peptide binding site and the ligand is oriented parallel to the β sheet of the PDZ domain, was selected for further refinement using explicit solvent MD simulations.

For each PDZ-inhibitor complex obtained from docking analysis, three independent explicit solvent MD simulations of 5 ns duration were carried out. Analysis of the binding modes revealed most of the low affinity (high K_i_ or K_d_) peptidomimectic ligands moved away from the peptide binding pocket, while ligands with high binding affinities (high K_i_ or K_d_) remained in the peptide binding pocket. Thus the differential selectivity of ligands by PDZ2 and PDZ3 domains could be explained by analysis of the final structures of the PDZ-inhibitor complexes obtained from MD simulations. Our analysis also revealed that, the differential specificities of the PDZ2 and PDZ3 domains primarily arise from differences in the conformation of the loop connecting βB and βC strands, because this loop interacts with the N-terminal chemical moieties of the ligands.

In order to make quantitative comparison with the experimental results, average MM/PBSA binding free energy was computed from the conformations sampled during last 1 ns of the 5 ns MD trajectories. MM/PBSA binding free energy was also computed for the conformation obtained from docking studies before MD simulations. For each of the two PDZ domains, the MM/PBSA binding free energies before and after MD simulations for all the 38 inhibitors were compared with the respective experimental binding free energy values. It was interesting to note that the MM/PBSA binding energy values showed a correlation coefficient of 0.56 and 0.569 with experimental binding free energy values for the PDZ2 and PDZ3 domains, respectively after exclusion of few outliers. However, the corresponding correlation coefficients for MM/PBSA binding energy values before MD was only 0.367 and 0.312, respectively. These results indicate that inclusion of complete flexibilities of the ligand and receptor helps in improving correlation with experimental results. It may be noted that in view of the challenges involved in quantitative prediction of binding affinities, the modest correlation between computational and experimental binding energies are indeed encouraging. Apart from the correlation between computational and experimental binding energies, our structural analysis has also revealed that despite having almost identical peptide binding pockets, PDZ2 and PDZ3 domains show differential selectivities for the same inhibitor, primarily because of the insertion of 6 residues in the loop between the β sheets βB and βC which interact with the N-terminal moieties of many inhibitors. Our structural analysis could also rationalize the effect of N-alkylation and mutations in tetrapeptide on experimental binding free energies. In summary, in this study we have successfully benchmarked a computational protocol for *in silico* identification of inhibitors of PDZ domains. Similar approach consisting docking, MD simulations and MM/PBSA binding energy calculation can be used for identifying small molecule or peptidomimetic inhibitors for other PRMs like PTB, WW and SH2 domains.

## Supporting Information

Figure S1 The workflow for exploring the binding modes of the known inhibitors of PDZ domains and calculation of their binding free energies.
(TIF)

Figure S2 Chemical structures of experimentally characterized inhibitors of PDZ domains. Each inhibitor is assigned a unique serial number. For serial numbers 1–36 : X = O or S; R = H (Ser) or CH_3_ (Thr); R_1_ = CH_3_ (Ala) or CH_2_COOH (Asp); R_2_ = CH_3_ (Ala) or CH_3_CH_2_COOH (Glu); R_3_ = (3,4-dichlorophenyl)ethyl, (naphthalene-2-yl)ethyl, Pyrenethyl, Pyrenbutyl or Trifluorophenylethyl. For serial numbers 37, 39–47 : R_1_ = −ph, −ph-4-Cl, −ph-4-Br, −ph-4-F, −ph-4-CH_3_, −ph-4-N(CH_2_CH_2_Cl)_2_, −ph-3,5-2(OCH_3_), -5-benzo[d] [1], [3]dioxol; R_2_ = −ph, −CH_3_, −ph-4-CH_3_, −ph-4-(OCH_3_), −ph-3,4,5-3(OCH_3_). For serial numbers 38,48 & 49 : R_1_ = −COOH, −CH_3_, −H; R_2_ = −CH_3_, −COOH; R_3_ = −CH_3_.
(TIF)

Figure S3 Superposition of the crystal structures of native peptide ligand bound PDZ domain of Shank protein on the inhibitor bound structure of the same PDZ domain.
(TIF)

Figure S4 (A) Bar graph showing the comparison of binding energy values determined experimentally as well as computed by using MM/PBSA after 5 ns of MD simulation for a set of 20 peptidomimetic ligands for both PDZ2 and PDZ3 domains. The ligand numbers given on x-axis is according to the numbering given in **Table 1**. (B) Bar graph showing the comparison of binding energy values determined experimentally as well as computed by using MM/PBSA after 5 ns of MD simulation for a set of 18 peptidomimetic ligands for both PDZ2 and PDZ3 domains. The ligand numbers given on x-axis is according to the numbering given in **Table 1**.
(TIF)

Figure S5 (A) Correlation between experimentally determined binding energy values and affinity values calculated from VINA for 2^nd^ PDZ domain of PSD-95 protein. (B) Correlation between experimentally determined binding energy values and affinity scores calculated from VINA for 3^rd^ PDZ domain of PSD-95 protein.
(TIF)

Table S1 Table summarizing binding energy values calculated by docking and MM/PBSA calculations for 38 docked ligand molecules on 2^nd^ PDZ domain of PSD-95 protein.
(PDF)

Table S2 Table summarizing binding energy values calculated by docking and MM/PBSA calculations for 38 docked ligand molecules on 3^rd^ PDZ domain of PSD-95 protein.
(PDF)
