# Supplementary figures and images for: An In Silico Analysis of the Binding Modes and Binding Affinities of Small Molecule Modulators of PDZ-Peptide Interactions
Source: PLoS One. 2013 Aug 8;8(8):e71340. doi: 10.1371/journal.pone.0071340 (PMC3738590; doi:10.1371/journal.pone.0071340)

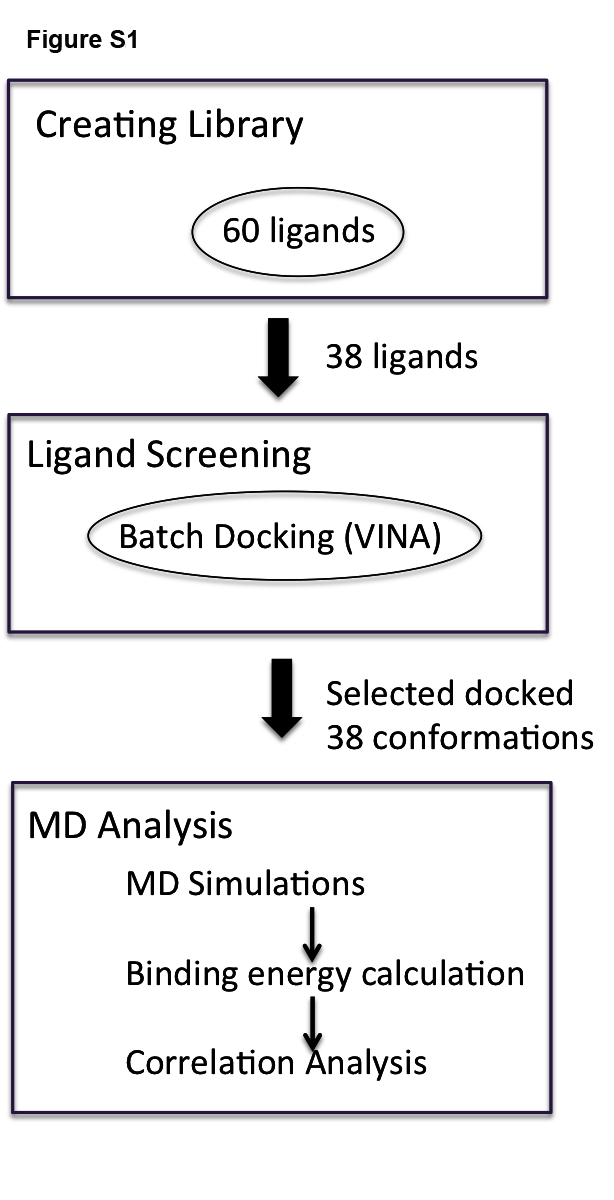

Supplement: Figure S1 — The workflow for exploring the binding modes of the known inhibitors of PDZ domains and calculation of their binding free energies. (TIF) [file pone.0071340.s001.tif]

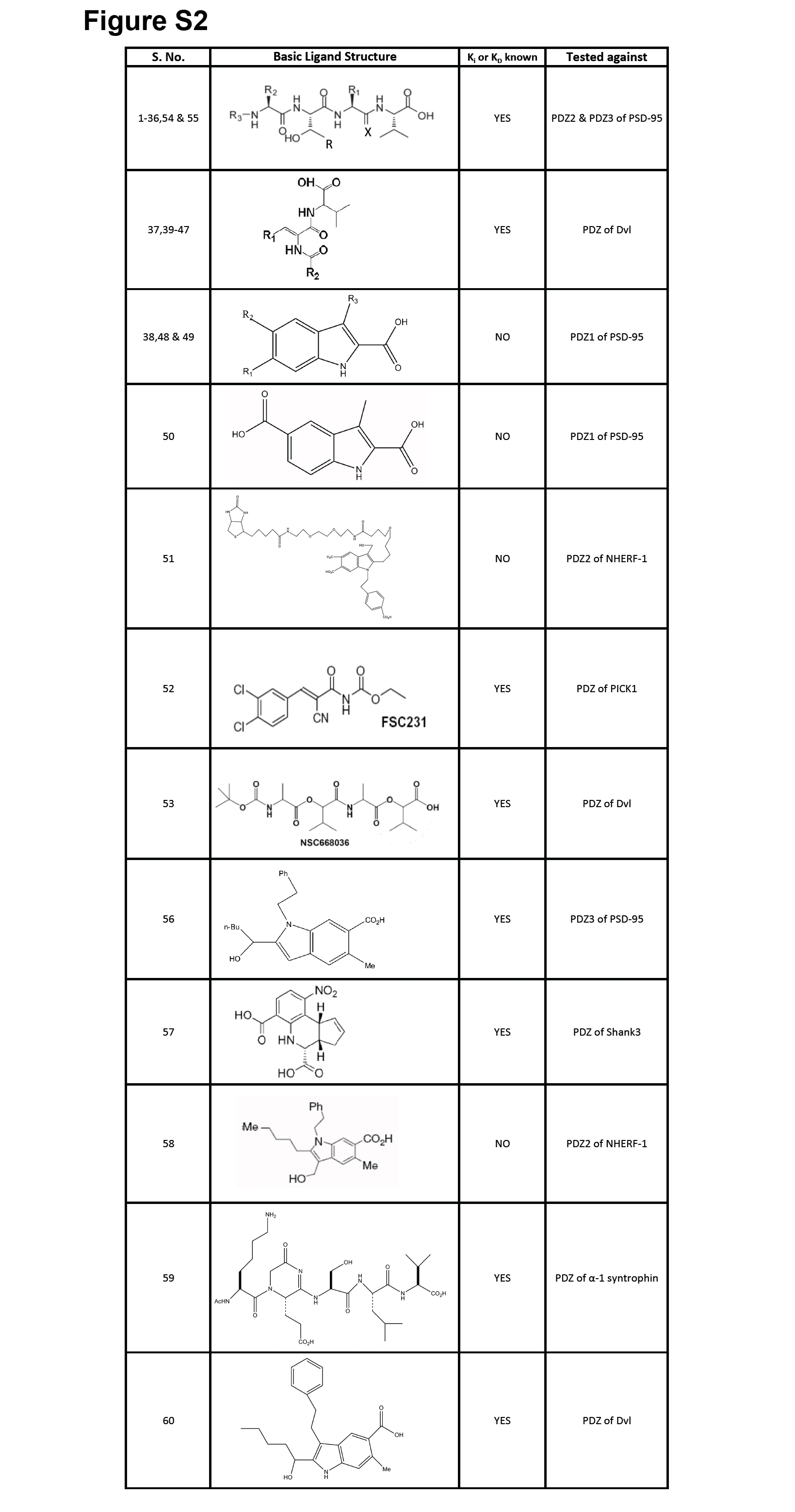

Supplement: Figure S2 — Chemical structures of experimentally characterized inhibitors of PDZ domains. Each inhibitor is assigned a unique serial number. For serial numbers 1–36 : X = O or S; R = H (Ser) or CH3 (Thr); R1 = CH3 (Ala) or CH2COOH (Asp); R2 = CH3 (Ala) or CH3CH2COOH (Glu); R3 = (3,4-dichlorophenyl)ethyl, (naphthalene-2-yl)ethyl, Pyrenethyl, Pyrenbutyl or Trifluorophenylethyl. For serial numbers 37, 39–47 : R1 = −ph, −ph-4-Cl, −ph-4-Br, −ph-4-F, −ph-4-CH3, −ph-4-N(CH2CH2Cl)2, −ph-3,5-2(OCH3), -5-benzo[d] [1], [3]dioxol; R2 = −ph, −CH3, −ph-4-CH3, −ph-4-(OCH3), −ph-3,4,5-3(OCH3). For serial numbers 38,48 & 49 : R1 = −COOH, −CH3, −H; R2 = −CH3, −COOH; R3 = −CH3. (TIF) [file pone.0071340.s002.tif]

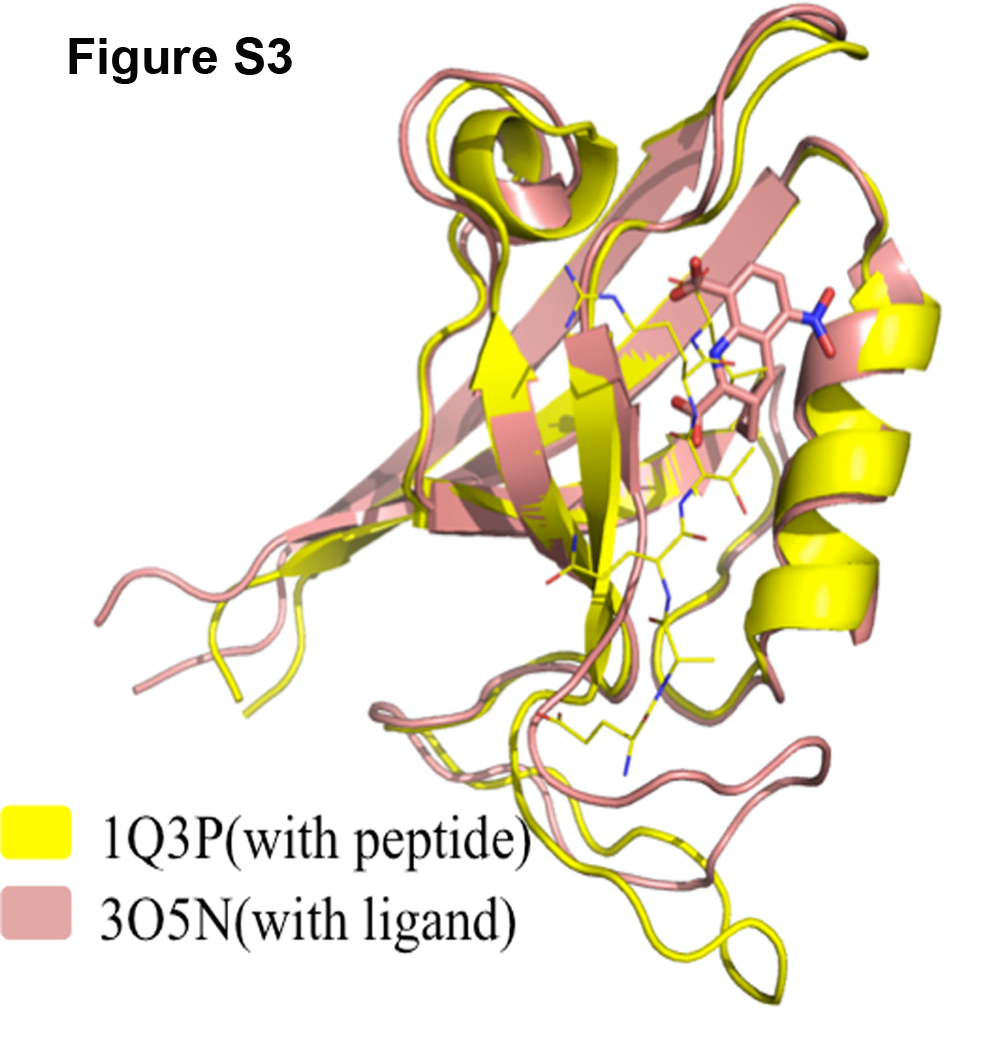

Supplement: Figure S3 — Superposition of the crystal structures of native peptide ligand bound PDZ domain of Shank protein on the inhibitor bound structure of the same PDZ domain. (TIF) [file pone.0071340.s003.tif]

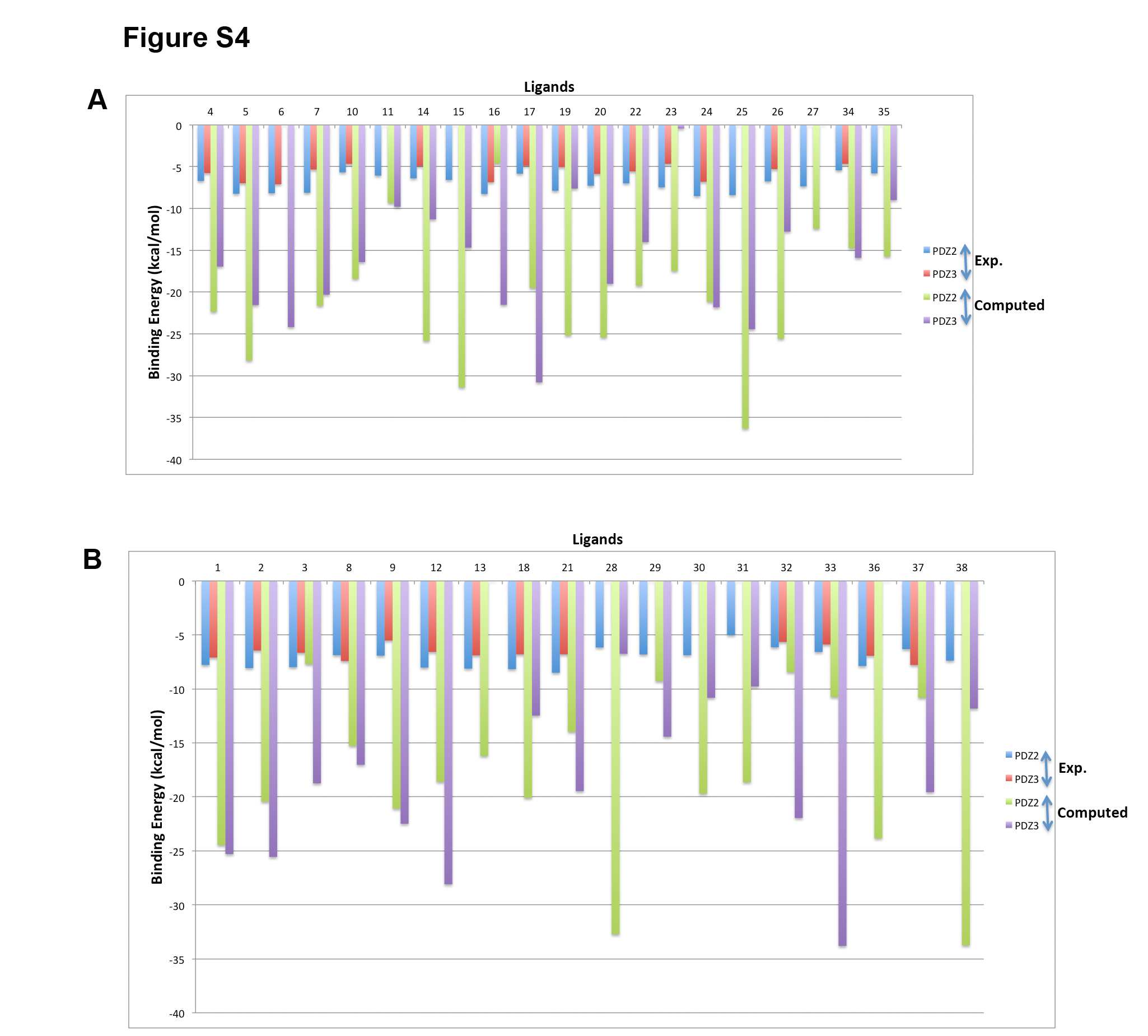

Supplement: Figure S4 — (A) Bar graph showing the comparison of binding energy values determined experimentally as well as computed by using MM/PBSA after 5 ns of MD simulation for a set of 20 peptidomimetic ligands for both PDZ2 and PDZ3 domains. The ligand numbers given on x-axis is according to the numbering given in Table 1 . (B) Bar graph showing the comparison of binding energy values determined experimentally as well as computed by using MM/PBSA after 5 ns of MD simulation for a set of 18 peptidomimetic ligands for both PDZ2 and PDZ3 domains. The ligand numbers given on x-axis is according to the numbering given in Table 1 . (TIF) [file pone.0071340.s004.tif]

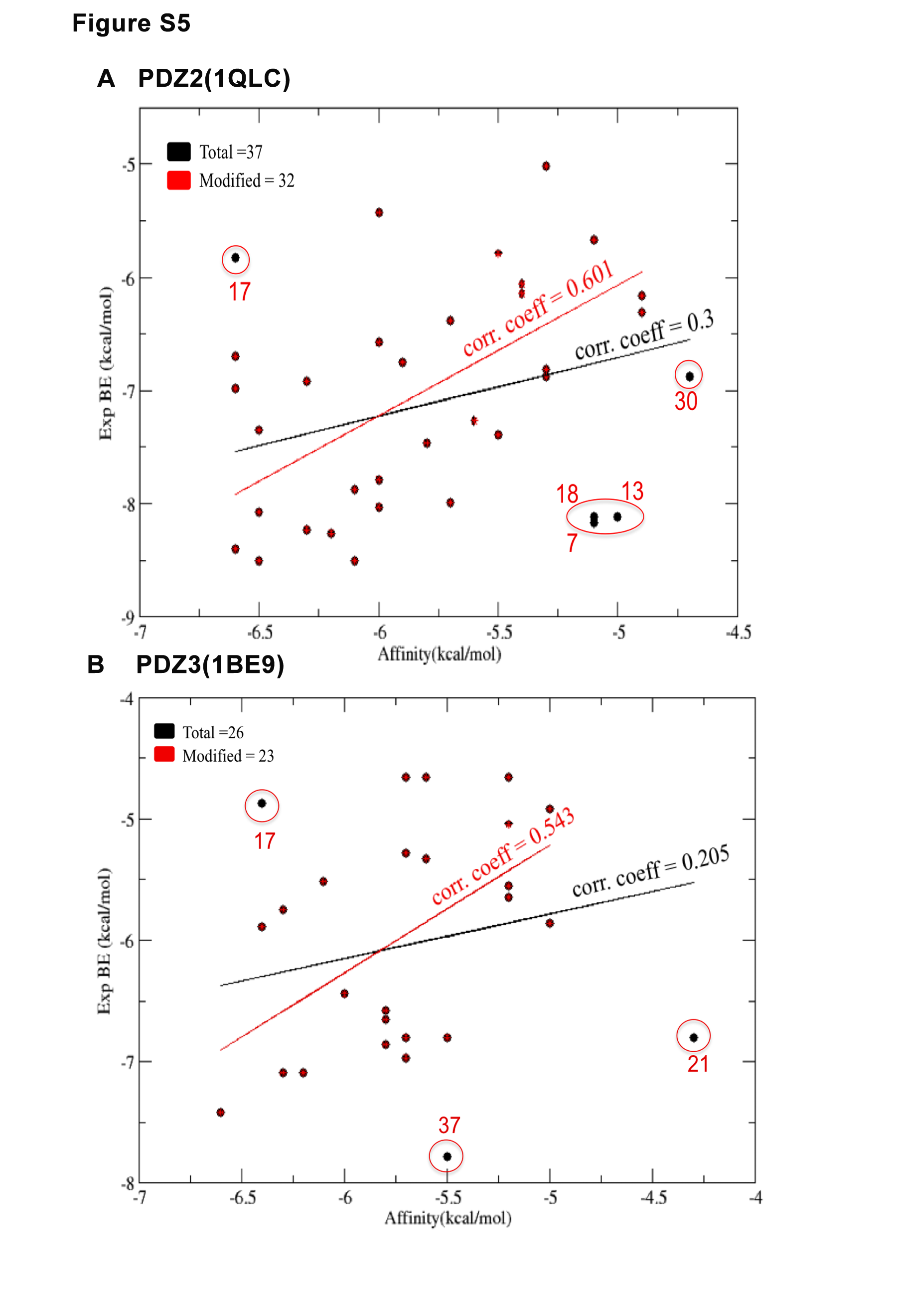

Supplement: Figure S5 — (A) Correlation between experimentally determined binding energy values and affinity values calculated from VINA for 2nd PDZ domain of PSD-95 protein. (B) Correlation between experimentally determined binding energy values and affinity scores calculated from VINA for 3rd PDZ domain of PSD-95 protein. (TIF) [file pone.0071340.s005.tif]
