# Supplementary material for: An In Silico Analysis of the Binding Modes and Binding Affinities of Small Molecule Modulators of PDZ-Peptide Interactions
Source: PLoS One. 2013 Aug 8;8(8):e71340. doi: 10.1371/journal.pone.0071340 (PMC3738590; doi:10.1371/journal.pone.0071340)
Supplement: Table S1 — Table summarizing binding energy values calculated by docking and MM/PBSA calculations for 38 docked ligand molecules on 2nd PDZ domain of PSD-95 protein. (PDF) [file pone.0071340.s006.pdf]

Table S1.Table summarizing binding energy values calculated by docking and MM/PBSA calculations for 38 docked ligand molecules on 2nd PDZ domain of PSD-95 protein.

S. No.	Sequence	R_3	X	PDZ2(1QLC)					
				Exp. K_i (μM)	BE (kcal/mol)	MODE	BE (VINA)	BE (MM/PBSA)	MM/PBSA (kcal/mol)[MD]
1	ETAV	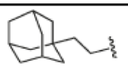	O	2.1(0.3)	-7.785	4th	-6	-32.67	-24.46
2	ETAV	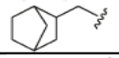	O	1.3(0.064)	-8.071	7th	-6.5	-18.7	-20.44
3	ETAV	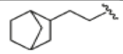	O	1.5(0.18)	-7.986	9th	-5.7	-24.24	-7.7
4	ATAV	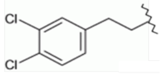	O	13(1.1)	-6.7	1st	-6.6	-35.41	-22.34
5	ETAV	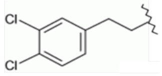	S	1.0(0.1)	-8.227	2nd	-6.3	-34.52	-28.18
6	ETAV	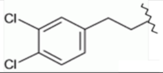	O	1.1(0.11)	-8.17	-	-	-	-
7	ETDV	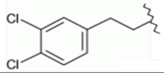	O	1.2(0.049)	-8.119	3rd	-5.1	-17.06	-21.63
8	ETAV	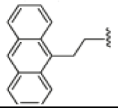	O	9.7(0.43)	-6.874	2nd	-5.3	-26.63	-15.27
9	ETAV	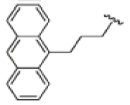	O	9.0(0.12)	-6.919	3rd	-6.3	-23.13	-21.08
10	ATAV	H	O	73(3.8)	-5.672	9th	-5.1	-13.72	-18.4
11	ATDV	H	O	38(4.2)	-6.061	5th	-5.4	-25.4	-9.33
12	ETAV	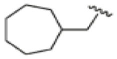	O	1.4(0.12)	-8.027	3rd	-6	-28.77	-18.6
13	ETAV	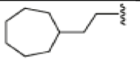	O	1.2(0.11)	-8.119	3rd	-5	-28.02	-16.2
14	ETAV	H	O	22(0.92)	-6.386	1st	-5.7	-22.59	-25.84
15	ETDV	H	O	16(0.88)	-6.576	3rd	-6	-40.03	-31.39

16	ETAV	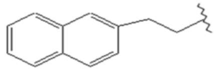	O	0.95(0.05)	-8.258	1st	-6.2	-11.49	-4.61
17	ATAV	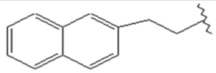	O	56(1.8)	-5.83	2nd	-6.6	-56.68	-19.51
18	ETAV	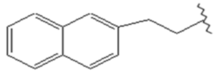	S	1.1(0.13)	-8.17	6th	-5.1	-17.25	-20.09
19	ETDV	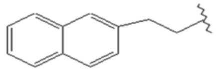	O	1.8(0.15)	-7.877	8th	-6.1	-30.85	-25.17
20	ETAV	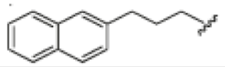	O	5.0(0.43)	-7.269	4th	-5.6	-54.96	-25.42
21	ETAV	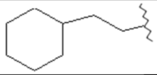	S	0.63(0.03)	-8.502	7th	-6.1	-18.74	-13.97
22	ATAV	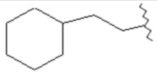	O	8.1(0.12)	-6.981	2nd	-6.6	-21.71	-19.21
23	ATDV	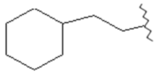	O	3.6(0.3)	-7.464	4th	-5.8	-19.09	-17.47
24	ETAV	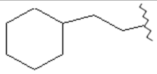	O	0.63(0.05)	-8.502	1st	-6.5	-40.27	-21.15
25	ETDV	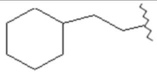	O	0.75(0.13)	-8.398	1st	-6.6	-38.07	-36.32
26	QTAV	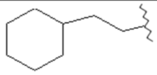	O	12(0.36)	-6.747	7th	-5.9	-34.88	-25.57
27	QTDV	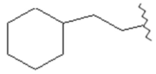	O	4.4(0.59)	-7.345	1st	-6.5	-28.51	-12.4
28	EsTAV	N-Me	O	32(2.8)	-6.163	2nd	-4.9	-29.5	-32.73
29	ETAV	N-Me	S	10.8(0.36)	-6.81	1st	-5.3	-0.45	-9.28
30	ETAV	N-Me	O	9.65(0.5)	-6.877	2nd	-4.7	-40.62	-19.7
31	ETsAV	N-Me	O	220(11)	-5.015	5th	-5.3	-8.46	-18.64
32	ETAV	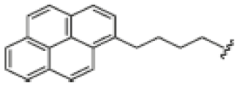	O	33(0.67)	-6.145	3rd	-5.4	-26.1	-8.45

33	ETAV	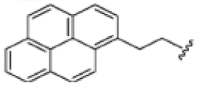	O	16(1.7)	-6.576	8th	-6	-19.46	-10.74
34	QTAV	H	O	110(6.3)	-5.428	1st	-6	-23.88	-14.73
35	QTDV	H	O	60(1.9)	-5.789	2nd	-5.5	-38.96	-15.73
36	ETAV	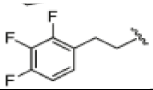	O	1.8(0.23)	-7.877	3rd	-6.1	-17.71	-23.83
37	KQTSV			25(1.6)	-6.31	7th	-4.9	-40.47	-10.82
38	IESDV			4.1(0.17)	-7.387	3rd	-5.5	-59.01	-33.75

***Fifth and sixth column indicates experimental Ki values and binding energy calculated from Ki. Seventh and eighth column indicates the rank and affinity predicted by VINA. Ninth column indicates binding energy value calculated using MM/PBSA taking minimized docked complex. Tenth column indicates binding energy value calculated using MM/PBSA from MD trajectory.**
