# Supplementary material for: An In Silico Analysis of the Binding Modes and Binding Affinities of Small Molecule Modulators of PDZ-Peptide Interactions
Source: PLoS One. 2013 Aug 8;8(8):e71340. doi: 10.1371/journal.pone.0071340 (PMC3738590; doi:10.1371/journal.pone.0071340)
Supplement: Table S2 — Table summarizing binding energy values calculated by docking and MM/PBSA calculations for 38 docked ligand molecules on 3rd PDZ domain of PSD-95 protein. (PDF) [file pone.0071340.s007.pdf]

Table S2. Table summarizing binding energy values calculated by docking and MM/PBSA calculations for 38 docked ligand molecules on 3rd PDZ domain of PSD-95 protein.

S. No.	Sequence	R_3	X	PDZ3(1BE9)					
				Exp. K_i (μM)	BE (kcal/mol)	MODE	BE (VINA)	BE (MM/PBSA)	MM/PBSA (kcal/mol)[MD]
1	ETAV	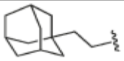	O	6.7±0.42	-7.094	4th	-6.2	-32.67	-25.3
2	ETAV	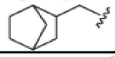	O	20±2.2	-6.443	4th	-6	-45.96	-25.56
3	ETAV	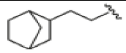	O	14±0.46	-6.656	4th	-5.8	-23.27	-18.75
4	ATAV	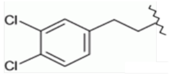	O	64±2.4	-5.751	1st	-6.3	-35.23	-16.94
5	ETAV	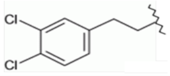	S	8.3±0.76	-6.967	3rd	-5.7	-22.54	-21.54
6	ETAV	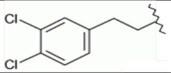	O	6.7±0.2	-7.094	2nd	-6.3	-24.04	-24.18
7	ETDV	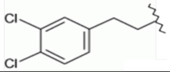	O	130±3.4	-5.329	1st	-5.6	-26.49	-20.3
8	ETAV	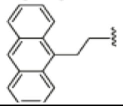	O	3.9±0.24	-7.417	1st	-6.6	-13.69	-17.03
9	ETAV	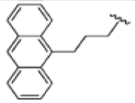	O	95±2.5	-5.515	3rd	-6.1	-14.72	-22.49
10	ATAV	H	O	>400	-4.659	2nd	-5.7	-24.79	-16.4
11	ATDV	H	O	-	-	4th	-4.2	-13.6	-9.79
12	ETAV	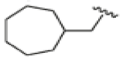	O	16±0.75	-6.576	3rd	-5.8	-35.13	-28.1
13	ETAV	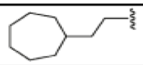	O	9.4±0.26	-6.893	-	-	-	-
14	ETAV	H	O	260±20	-4.916	4th	-5	-18.25	-11.29
15	ETDV	H	O	-	-	2nd	-5.2	-25.28	-14.68

16	ETAV	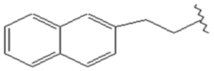	O	10±0.22	-6.856	8th	-5.8	-23.73	-21.52
17	ATAV	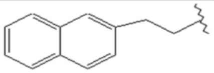	O	280±9.0	-4.872	2nd	-6.4	-51.82	-30.79
18	ETAV	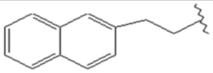	S	11±0.44	-6.799	1st	-5.7	-17.57	-12.47
19	ETDV	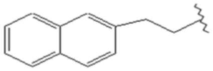	O	210±8.5	-5.043	8th	-5.2	-19.25	-7.61
20	ETAV	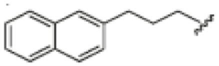	O	53±4.0	-5.863	2nd	-5	-29.18	-19
21	ETAV	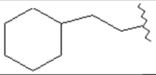	S	11±0.97	-6.799	7th	-4.3	-15.25	-19.47
22	ATAV	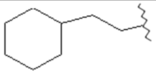	O	89±3.3	-5.554	7th	-5.2	-26.13	-14.01
23	ATDV	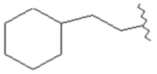	O	>400	-4.659	1st	-5.6	-9.28	-0.42
24	ETAV	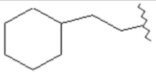	O	11±0.79	-6.799	1st	-5.5	-28.3	-21.81
25	ETDV	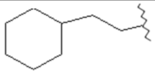	O	-	-	5th	-5.1	-39.07	-24.43
26	QTAV	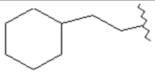	O	141±14	-5.28	6th	-5.7	-15.68	-12.76
27	QTDV	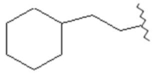	O	-	-	-	-	-	-
28	EsTAV	N-Me	O	-	-	4th	-4.8	-11.07	-6.75
29	ETAV	N-Me	S	-	-	3rd	-4.7	-15.57	-14.43
30	ETAV	N-Me	O	-	-	2nd	-4.8	-17.46	-10.82
31	ETsAV	N-Me	O	-	-	9th	-4.8	-21.27	-9.77
32	ETAV	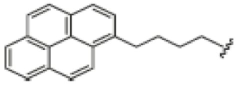	O	76±4.9	-5.648	8th	-5.2	-27.42	-21.96

33	ETAV	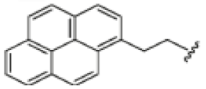	O	51±4.3	-5.886	3rd	-6.4	-29.59	-33.81
34	QTAV	H	O	>400	-4.659	8th	-5.2	-27.52	-15.9
35	QTDV	H	O	-	-	9th	-5	-28.81	-8.98
36	ETAV	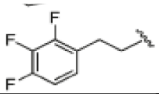	O	8.8±0.63	-6.932	-	-	-	-
37	KQTSV			2.1±0.15	-7.785	1st	-5.5	-30.35	-19.56
38	IESDV			-	-	2nd	-5.6	-40.77	-11.82

***Fifth and sixth column indicates experimental Ki values and binding energy calculated from Ki. Seventh and eighth column indicates the rank and affinity predicted by VINA. Ninth column indicates binding energy value calculated using MM/PBSA taking minimized docked complex. Tenth column indicates binding energy value calculated using MM/PBSA from MD trajectory.**
